# Standardization of electrolyte leakage data and a novel liquid nitrogen control improve measurements of cold hardiness in woody tissue

**DOI:** 10.1186/s13007-021-00755-0

**Published:** 2021-05-22

**Authors:** Alisson P. Kovaleski, Jake J. Grossman

**Affiliations:** 1 Arnold Arboretum of Harvard University, 1300 Centre St., Boston, MA 02131 USA; 2 Department of Horticulture, University of Wisconsin-Madison, 1575 Linden Drive, Madison, WI 53706 USA; 3 Biology Department, Swarthmore College, 500 College Ave., Swarthmore, PA 19081 USA

**Keywords:** Acer, Cold hardiness, Differential thermal analysis, Electrolyte leakage, Freezing tolerance, Maple

## Abstract

**Background:**

A variety of basic and applied research programs in plant biology require the accurate and reliable determination of plant tissue cold hardiness. Over the past 50 years, the electrolyte leakage method has emerged as a popular and practical method for quantifying the amount of damage inflicted on plant tissue by exposure to freezing temperatures. Numerous approaches for carrying out this method and analyzing the resultant data have emerged. These include multiple systems for standardizing and modeling raw electrolyte leakage data and multiple protocols for boiling or autoclaving samples in order to maximize leakage as a positive control. We compare four different routines for standardization of leakage data and assess a novel control method—immersion in liquid nitrogen in lieu of traditional autoclaving—and apply them to woody twigs collected from 12 maple (*Acer*) species in early spring. We compare leakage data from these samples using each of four previously published forms of data analysis and autoclaving vs. liquid nitrogen controls and validate each of these approaches against visual estimates of freezing damage and differential thermal analysis.

**Results:**

Through presentation of our own data and re-analysis of previously published findings, we show that standardization of raw data against estimates of both minimum and maximum attainable freezing damage allows for reliable estimation of cold hardiness at the species level and across studies in diverse systems. Furthermore, use of our novel liquid nitrogen control produces data commensurate across studies and enhances the consistency and realism of the electrolyte leakage method, especially for very cold hardy samples.

**Conclusion:**

Future leakage studies that relativize data against minimum and maximum leakage and that employ our updated liquid nitrogen control will contribute generalizable, repeatable, and realistic data to the existing body of cold hardiness research in woody plants. Data from studies conducted using a liquid nitrogen (and not an autoclaving) control can still be compared to previously published data, especially when raw data are standardized using the best-performing approach among those we assessed. Electrolyte leakage of woody twigs emerges as a useful technique for quickly assessing the probability of tissue death in response to freezing in dormant plants. Differential thermal analysis may provide different and complementary information on cold hardiness.

**Supplementary Information:**

The online version contains supplementary material available at 10.1186/s13007-021-00755-0.

## Background

How do plants differ in their cold hardiness, the ability to avoid or tolerate exposure to temperatures below freezing [1]? The capacity to withstand freezing temperatures structures global plant distribution, determining which lineages can radiate into high-latitude or -altitude environments [2, 3]. Within these environments, plants that can withstand not just freezing, but extreme cold, are able to realize a larger niche than those more vulnerable to frost or freezing [4, 5]. In short, cold hardiness shapes the ecological dynamics and evolutionary trajectory of temperate plant evolution. Across research domains, researchers require tools for evaluating cold hardiness that can be efficiently repeated across diverse plant lineages while providing interpretable, mechanistic evaluations of cold hardiness traits.

In the years since 1932 when Dexter and colleagues’ [6] observation that freezing temperatures destabilize the cellular membrane and accelerate leakage of symplastic solutes out of the cell, quantification of *electrolyte leakage* has emerged as a tool for quantifying cold hardiness. Flint et al. [7] subsequently developed the Index of Injury (*I*) approach, which is calculated through comparison of samples incubated in pure water after being frozen. Values of *I* close to 0 indicate very little disruption of cellular stability and consequently little leakage while higher values indicate greater disruption and more leakage. Over the past 50 years, the measurement of electrolyte leakage from stem and leaf tissues of woody plants adapted to cold climates has remained popular (reviewed in the Introduction of [8]; see [9–11] for more recent examples). Despite this, there exists considerable diversity in the benchtop and analytical approaches employed in electrolyte leakage measurements. We carried out the present work with a particular focus on variability in two areas: the analytical procedures for extraction of critical cold hardiness values from electrolyte leakage data and the control treatment used to relativize freezing damage.

### How is cold hardiness determined from leakage?

**Fig. 1:**
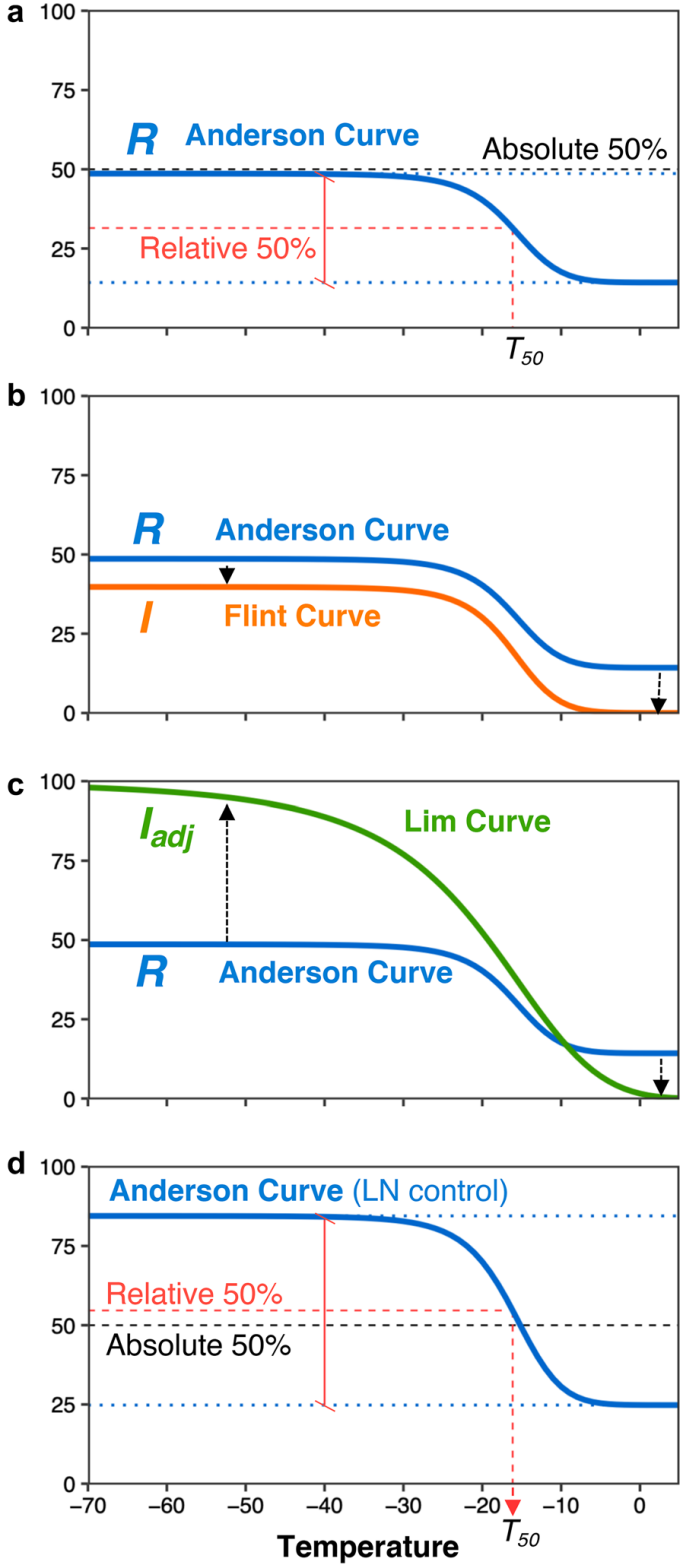
Schematic illustration comparing different approaches to measuring electrolyte leakage. **A** In the Anderson approach (no minimum or maximum leakage specified, yields *R*), samples may not reach an “Absolute 50%” damage point. Instead, the temperature at which “Relative 50%” damage is attained may be more meaningful. **B** By comparison, values of *I,* in the Flint approach (orange line) are zeroed, although this may not drastically displace the curve relative to an Anderson curve. **C** In the Lim approach, data are stretched between 0 and 100% damage, usually at the warmest and coldest temperature treatments, respectively*.* **D** Use of a liquid nitrogen control is expected to elevate all leakage values, making, for instance, an Anderson curve behave more like a Lim curve (“Absolute” and “Relative” 50% values similar), and improving generalizability among approaches

Since the development of the electrolyte leakage method, practitioners have employed diverse approaches to determine cold hardiness from raw electrolyte leakage data. Typically, procedures follow a three-step approach: absolute electrolyte leakage of a sample is made relative (*R*) by normalizing against electrolyte leakage at an extreme control (typically boiling or autoclaving, as discussed further below), followed by optional adjustment that results in *I,* and curve fitting, which allows extraction of a critical cold hardiness value. Variation in the choices made at each of these steps has produced variation in the ways that leakage data are interpreted in the literature (Fig. 1).

At the first stage of processing electrolyte leakage data, once *R* is calculated for a given individual across a range of freezing temperatures, it can be transformed into *I* by “zeroing” against unfrozen control samples and *I*_*adj*_ by also “stretching” relative to a theoretical maximum level of damage. In their work, Anderson et al. [12] opt to do neither; they simply fit curves of *R* (EL in their notation) vs. freezing temperature across a wide range of temperatures (5 to – 196 ℃). In this case, *R* can range from 0 to 100%, but these values are rarely attained. Flint and colleagues’ [7] approach is similar, except that *R* values are zeroed against a negative control kept at room temperature to take into account any electrolyte leakage from samples that might occur due to handling and processing unrelated to freezing (e.g., cut ends of stem pieces), thus becoming *I*. Curves fit to these data therefore treat freezing damage commensurate with handling and processing damage as “0%” damage by default. Finally, Lim and colleagues [13], popularized an approach that goes one step further, stretching all values of *I* prior to curve fitting such that a sample’s leakage at the lowest temperature tested (often at or below – 80 ℃) is treated as the maximum possible damage for that sample, giving *I*_*adj*_. Curves fit using this procedure contain values of *I* corresponding to 0 and 100% damage. These three approaches represent a gradient in adjustment of raw electrolyte leakage values ranging from none (“Anderson”) to zeroing against a minimum (“Flint”) to both zeroing and stretching to a maximum (“Lim”).

Second, curves are fit to adjusted data to model progressive accumulation of damage with exposure to freezing. Historically, most authors have fit data to generalized logistic curves [12, 14–20], but some may use linear models as well [11]. However, while such logistic models always represent damage in response to dropping temperatures as a symmetric, sigmoidal process, actual plant tissues may accrue damage asymmetrically. This is to say, for instance, that marginal increases in damage may occur much more rapidly (over smaller temperature intervals) prior to the inflection point of the sigmoid curve than after it. For this reason, Von Fircks and Verwijst [21] first suggested using the Richards function, which can produce asymmetric, sigmoidal curves, to model *I* over a range of temperatures. Subsequent work [8] has validated this approach, with Lim and colleagues [13] recommending the Gompertz function—a special case of the Richards with one fewer parameter—as the best fit for leakage data. Many subsequent workers have followed their example and modeled leakage data with the Gompertz function [9, 22–24]. As such data processed using the Anderson, Flint, or Lim approach can then be modeled within either a logistic or Gompertz framework.

Third, and finally, a critical value of freezing damage, used to approximate cold hardiness, is extracted from model fit to leakage data. For general logistic models, this usually corresponds to *T*_*50*_, the temperature at which 50% of maximum possible electrolyte leakage is attained, although other damage thresholds can be adopted as well (eg., *T*_*20*_, *T*_*80*_, etc.). Yet modeling of leakage data with the asymmetric Richards and Gompertz curves has also made it possible to extract a potentially more biologically realistic critical value from the resulting fitted curves. Whereas the inflection point of a logistic curve, *T*_*max*_, always corresponds with *T*_*50*_, these two points are decoupled in asymmetric sigmoid curves. As such, *T*_*max*_, the temperature at which the rate of increase in freezing-induced damage is maximized (the temperature of maximum instantaneous slope of the curve), does not have to co-occur with the temperature at which half of the maximum damage possible has been reached. And though *T*_*max*_ has been employed in place of *T*_*50*_ in the literature [8, 13] validation of this choice with reference to other cold hardiness metrics has been limited. As such, differences in the biological realism and thus desirability of the two critical values are still unclear.

### What is the best way to standardize measurements of freezing-induced leakage?

Electrolyte leakage data have historically been standardized at the level of the individual sample to control for variability among samples in size and intrinsic (e.g., species- or genotype-dependent) electrolyte leakage capacity. In this work, we also assess the realism and consistency of the most commonly used positive control technique. Typically, all samples are heat-killed [25] following the first conductivity reading, often through autoclaving. Yet, as a control for measurements of cold hardiness, heat killing fails to mimic the process of interest: freezing-induced damage. In this vein, at least two groups [8, 13] have used conductivity following freezing at – 80 ℃ in lieu of heat killing as an index of maximum leakage following damage to sampled tissues. However, it has remained unclear whether freezing at these temperatures is actually sufficient to relativize leakage across samples varying in size and anatomy [26].

Furthermore, the temperature at which samples are boiled or heated and/or the time during which this temperature is reached are frequently not specified ([10, 12, 17, 27–29], among others). And though autoclaving at 120/121 ℃ is typical (e.g., [9, 20, 30]), some [31–34] report using temperatures lower than the boiling point of water to heat kill samples. Yet Deans et al. [16] find that even temperatures above 100 ℃ (they compare 105 vs. 121 ℃) vary in their capacity to induce electrolyte leakage. This variability may be especially problematic for woody stem tissue (as opposed to bud or dissected vascular samples), from which leakage may be constrained to cut ends rather than from sample’s entire surface area. In short, because electrolyte leakage depends on the temperature of and duration of and time elapsing following heat-killing [16, 35], boiling or autoclaving as a control strikes us as a frequent and consequential source of inconsistency in the method.

### Our approach

Despite some helpful methodological comparisons [13, 16, 22], practitioners collecting electrolyte leakage data are still confronted with numerous choices: what type of standardizing control should be employed during data collection? Should data be analyzed using the Anderson, Flint, or Lim approach to zeroing and stretching? Which critical value should be extracted? And, once such decisions are made, will the resulting estimates of cold hardiness actually predict field plant performance?

In the work presented here, we respond to existing diversity in the methods used to assess cold hardiness through electrolyte leakage by: (i) integrating novel measurements in a panel of 12 maple (*Acer* spp.) species (Additional file 1: Figure S1), and (ii) re-analyzing published data. Our aim is to consolidate existing methods in measuring leakage, validate these methods against other techniques for measuring cold hardiness, and suggest a standard operating procedure in contrast to the variable status quo. We focus on two main domains in which existing practice could be improved. First, we contrast the performance of four main approaches to processing maple electrolyte leakage data: logistic modeling of data adjusted following (1) Anderson et al. [12], (2) Flint et al. [7], and (3) Lim et al. [13] and (4) Gompertz modeling of data adjusted following the Lim approach. And second, we introduce a novel control procedure of liquid nitrogen immersion and compare its performance to that of the standard autoclaving control. Our approach addresses four questions:

1. Which of the four approaches to modeling electrolyte leakage data produce estimates of cold hardiness most aligned with those from two other core approaches: visual damage and differential thermal analysis?
2. Does use of a liquid nitrogen control
  - produce leakage data commensurate with those generated with an autoclaving control?
  - improve generalizability of findings across approaches to modeling leakage data?
3. Does re-analysis of previously collected data [10, 11] using data zeroing and stretching and assuming damage commensurate with a liquid nitrogen control provide more realistic estimates of cold hardiness?

In presenting this analysis, we encourage other investigators to continue using electrolyte leakage to measure cold hardiness and facilitate synthesis of existing and forthcoming data despite the use of diverse protocols.

## Results

### Electrolyte leakage data zeroed and stretched using the Lim approach best approximated visual damage to maple twigs

**Fig. 2:**
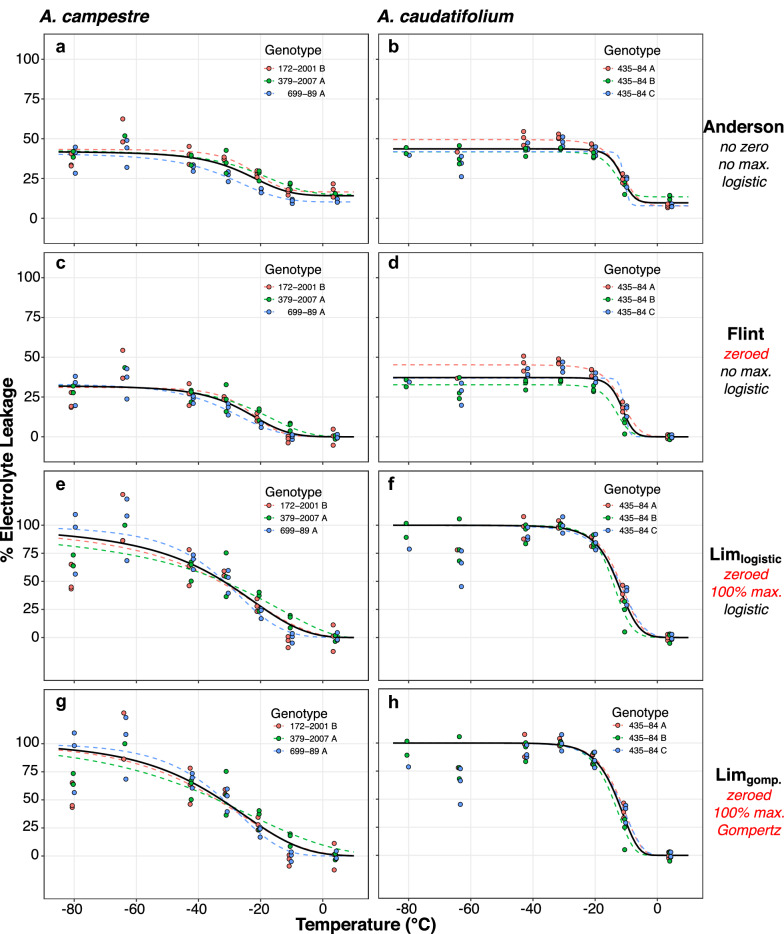
Comparison of four approaches for fitting curves to data representing the relationship between freezing damage and temperature in (**A**, **C**, **E**, **G**) *A. caudatifolium* and (**B**, **D**, **F**, **H**) *A. campestre* stem segments (plots for other species provided in Additional file 1). Curves fit to data on a per-genotype (red, blue, and green) and per-species (black curve) basis are fit in each case. Panels show curves fit following the approach of **A**, **B** Anderson et al. [12], **C**, **D** Flint et al.[7], and Lim et al. [13]. Approaches vary, as indicated, in their use of room-temperature (zeroing; **C**–**H**) and deep freezing (maximum damage; **E**–**H**) controls and reliance on general logistic (**A**–**F**) vs. Gompertz (**G**–**H**) curves

By design, the four analytical approaches that we compared generated variability in curves representing the relationship between electrolyte leakage (*R* or *I*) and freezing temperature (Fig. 2; model parameters given in Additional file 2A: Table S1). Yet critical values extracted using each approach occupy a similar range of values (− 12 to − 28 ℃; Table 1A) and were generally correlated with each other (Table 2); species that had already broken flower or leaf buds were generally less cold hardy than those that still appeared dormant (Table 1A). Across methods, leakage measurements generated a warmer and somewhat narrower range of critical values relative to visual damage estimates (− 17 to − 36 ℃) and LTEs (− 20 to − 38 ℃, Table 1A).

**Table 1:** Phenological condition and cold hardiness of (A) maple and (B) oak species

Species	Condition	Critical Values for Electrolyte Leakage					Visual LT_50_, ℃	LST, ℃	LTE, ℃
		Anderson	Flint	Lim_logistic_	Lim_gompertz_	Lim_gompertz_			
		LT_50_, ℃	LT_50_, ℃	LT_50_, ℃	LT_50_, ℃	LT_max_, ℃			
(A). Maple (*Acer*) data from the present study									
*A. saccharum*	Dormant	− 28^b^	− 28^b^	− 26^ cd^	− 26^ cd^	− 21^bc^	− 29^ab^	− 33^ab^	− 36^bc^
*A. pseudoplatanus*	Dormant	− 20^ab^	− 20^ab^	− 22^bc^	− 22^bcd^	− 18^abc^	− 30^ab^	− 33^ab^	− 38^ cd^
*A. rubrum*	Budbreak	− 16^ab^	− 16^ab^	− 17^ab^	− 17^ab^	− 14^ab^	− 18^a^	− 17^a^	− 35^b^
*A. platanoides*	Dormant	− 25^ab^	− 24^ab^	− 25^bcd^	− 25^bcd^	− 20^bc^	− 27^ab^	− 30^ab^	− 35^b^
*A. campestre*	Dormant	− 25^ab^	− 25^b^	− 32^d^	− 33^d^	− 26^c^	− 36^b^	− 40^b^	− 37^ cd^
*A. hyrcanum*	Dormant	− 23^b^	− 23^ab^	− 23^bc^	− 23^bcd^	− 18^abc^	− 30^ab^	− 33^ab^	− 36^ab^
*A. tataricum*	Bud swelling	− 18^ab^	− 18^ab^	− 19^abc^	− 19^abc^	− 14^ab^	− 19^a^	− 20^ab^	− 36^bcd^
*A. tegmentosum*	Budbreak	− 18^ab^	− 17^ab^	− 18^abc^	− 18^abc^	− 14^ab^	− 17^a^	− 23^ab^	− 36^bcd^
*A. caudatifolium*	Budbreak	− 12^a^	− 12^a^	− 13^a^	− 13^a^	− 11^a^	− 20^a^	− 20^ab^	− 20^a^
*A. davidii*	Bud swelling	− 17^ab^	− 18^ab^	− 19^abc^	− 20^abc^	− 16^ab^	− 21^a^	− 27^ab^	− 20^a^
*A. spicatum*	Dormant	− 20^ab^	− 21^ab^	− 20^abc^	− 21^abc^	− 16^ab^	− 27^ab^	− 23^ab^	− 38^d^
*A. negundo*	Dormant	− 27^b^	− 27^b^	− 24^bcd^	− 26^bcd^	− 18^abc^	− 20^a^	− 23^ab^	− 36^bcd^

(B). Oak (*Quercus*) data from Fallon and Cavender-Bares (2018)							Published LT_50_, ℃	N
*Q. arizonica*	Acclimated	− 22^b^	− 21^bc^	− 21^bc^	− 21^bc^	− 18^b^	− 34	39
	Unacclimated	− 12^a^	− 12^a^	− 12^a^	− 12^a^	− 10^a^	− 16	44
*Q. emoryi*	Acclimated	− 26^c^	− 26^d^	− 25^d^	− 26^d^	− 23^c^	− 29	12
	Unacclimated	− 14^a^	− 14^a^	− 14^a^	− 14^a^	− 11^a^	− 15	10
*Q. gambelii*	Acclimated	− 22^b^	− 21^bc^	− 21^bc^	− 21^bc^	− 18^b^	− 33	17
	Unacclimated	− 11^a^	− 11^a^	− 12^a^	− 12^a^	− 10^a^	− 20	16
*Q. grisea*	Acclimated	− 25^bc^	− 25^ cd^	− 25^ cd^	− 26^ cd^	− 23^c^	Not calculated	2
	Unacclimated	− 16^ab^	− 15^ab^	− 15^ab^	− 15^ab^	− 12^ab^	Not calculated	2
*Q. hypoleucoides*	Acclimated	− 22^b^	− 21^bc^	− 21^bc^	− 21^bc^	− 18^b^	− 23	39
	Unacclimated	− 13^a^	− 13^a^	− 13^a^	− 13^a^	− 11^a^	− 13	40
*Q. rugosa*	Acclimated	− 24^bc^	− 23^ cd^	− 23^ cd^	− 23^ cd^	− 19^bc^	− 32	16
	Unacclimated	− 13^a^	− 13^a^	− 13^a^	− 13^a^	− 11^a^	− 17	17

Phenological condition at sampling and critical values of cold hardiness for electrolyte leakage, lowest survival temperatures (LSTs), and low temperature exotherms (LTEs) for A) maple species (n = 3) and B) oak species-acclimation combinations (sample size and originally published critical values as noted; Fallon and Cavender-Bares, 2018). In cases for which differences among species are significant, superscripts indicate results of a Tukey's HSD post-hoc test (α = 0.05). Samples vary in phenological condition at the time of sampling. Maples were sampled at various points ranging from dormancy to post-budbreak in early spring. Oaks were sampled while either cold-acclimated (winter) or unaccclimated (summer)

**Table 2:** Correlations between critical values of cold hardiness based on different approaches

		Anderson			Flint			Lim_logistic_			Lim_gompertz_				Visual damage			
		LT_20_	LT_50_	LT_80_	LT_20_	LT_50_	LT_80_	LT_20_	LT_50_	LT_80_	LT_20_	LT_50_	LT_80_	LT_max_	LT_20_	LT_50_	LT_80_	LST
Anderson	LT_20_																	
	LT_50_	0.44																
	LT_80_	− 0.10	**0.84**															
Flint	LT_20_	**0.92**	**0.54**	0.60														
	LT_50_	0.40	**Identical**	**0.85**	**0.53**													
	LT_80_	− 0.30	**0.88**	**0.99**	0.10	**0.89**												
Lim_logistic_	LT_20_	**0.89**	**0.60**	0.11	**0.94**	**0.59**	0.17											
	LT_50_	**0.59**	**0.81**	**0.50**	**0.72**	**0.81**	**0.55**	**0.74**										
	LT_80_	**0.20**	**0.70**	**0.63**	**0.34**	**0.72**	**0.65**	**0.33**	**0.88**									
Lim_gompertz_	LT_20_	**0.91**	0.48	− 0.20	**0.98**	0.47	0.30	**0.96**	**0.69**	**0.29**								
	LT_50_	**0.52**	**0.82**	**0.56**	**0.65**	**0.83**	**0.60**	**0.68**	**Identical**	**0.91**	**0.62**							
	LT_80_	**0.24**	**0.78**	**0.68**	**0.37**	**0.80**	**0.72**	**0.41**	**0.91**	**0.98**	**0.33**	**0.94**						
	LT_max_	**0.70**	**0.78**	**0.41**	**0.83**	**0.78**	**0.46**	**0.84**	**0.98**	**0.78**	**0.81**	**0.96**	**0.82**					
Visual Damage	LT_20_	**0.28**	0.28	0.13	**0.34**	0.30	0.16	**0.35**	**0.42**	0.34	**0.34**	**0.40**	0.34	**0.42**				
	LT_50_	**0.44**	**0.46**	0.22	**0.50**	**0.47**	0.27	**0.51**	**0.60**	**0.49**	**0.49**	**0.58**	**0.50**	**0.61**	**0.85**			
	LT_80_	**0.46**	**0.49**	0.24	**0.51**	**0.50**	0.30	**0.53**	**0.62**	**0.49**	**0.50**	**0.60**	**0.51**	**0.62**	**0.58**	**0.92**		
	LST	**0.49**	**0.43**	0.14	**0.51**	**0.43**	0.21	**0.54**	**0.59**	**0.46**	**0.52**	**0.57**	**0.47**	**0.61**	**0.48**	**0.80**	**0.89**	
LTE		− **0.31**	− **0.64**	− **0.58**	− **0.31**	− **0.63**	− **0.62**	− **0.36**	− **0.46**	− **0.45**	− 0.27	− **0.47**	− **0.48**	− **0.43**	− 0.26	− **0.34**	− **0.35**	− **0.28**

Correlations (⍴) between critical values of cold hardiness (℃) for four electrolyte leakage approaches, visual damage (including lowest survival temperature), and low temperature exotherms. Correlations are calculated for maple genotype means (n = 36) for all indices except LTEs, for which species means (n = 12) are compared to other metrics. We focus on the use of the LT50 critical value extracted using the Lim_logistic_ approach (enclosed with borders). Bolded correlations indicate a significant (α < 0.05) bivariate correlation test

**Fig. 3:**
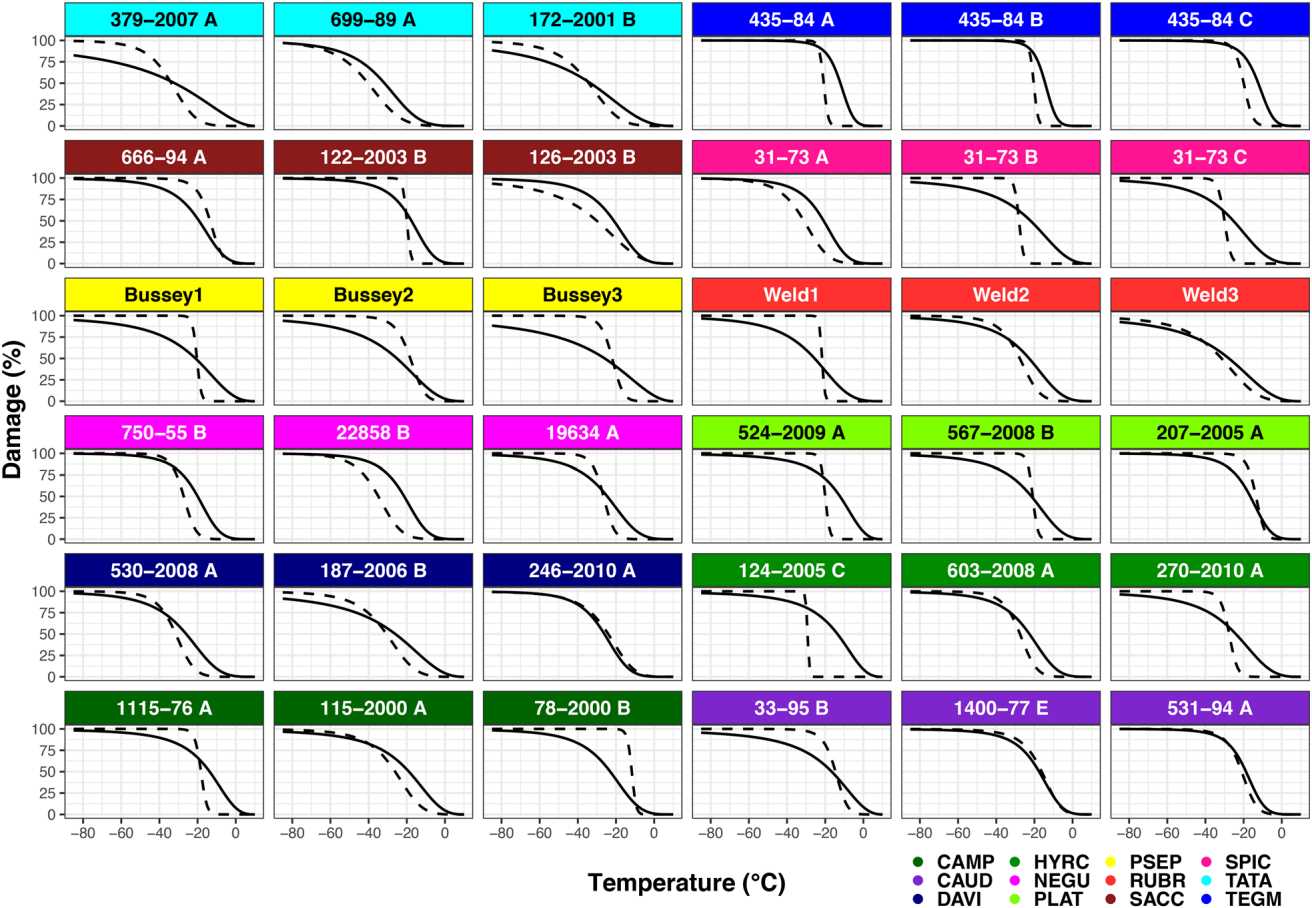
Damage, as reflected by electrolyte leakage (solid lines) and visual estimates (dashed lines), induced by freezing from − 10 to 80 ℃. Electrolyte leakage is calculated using the Lim_logistic_ approach. Panels represent estimates of damage to particular genotypes. Color-coding indicates species

**Fig. 4:**
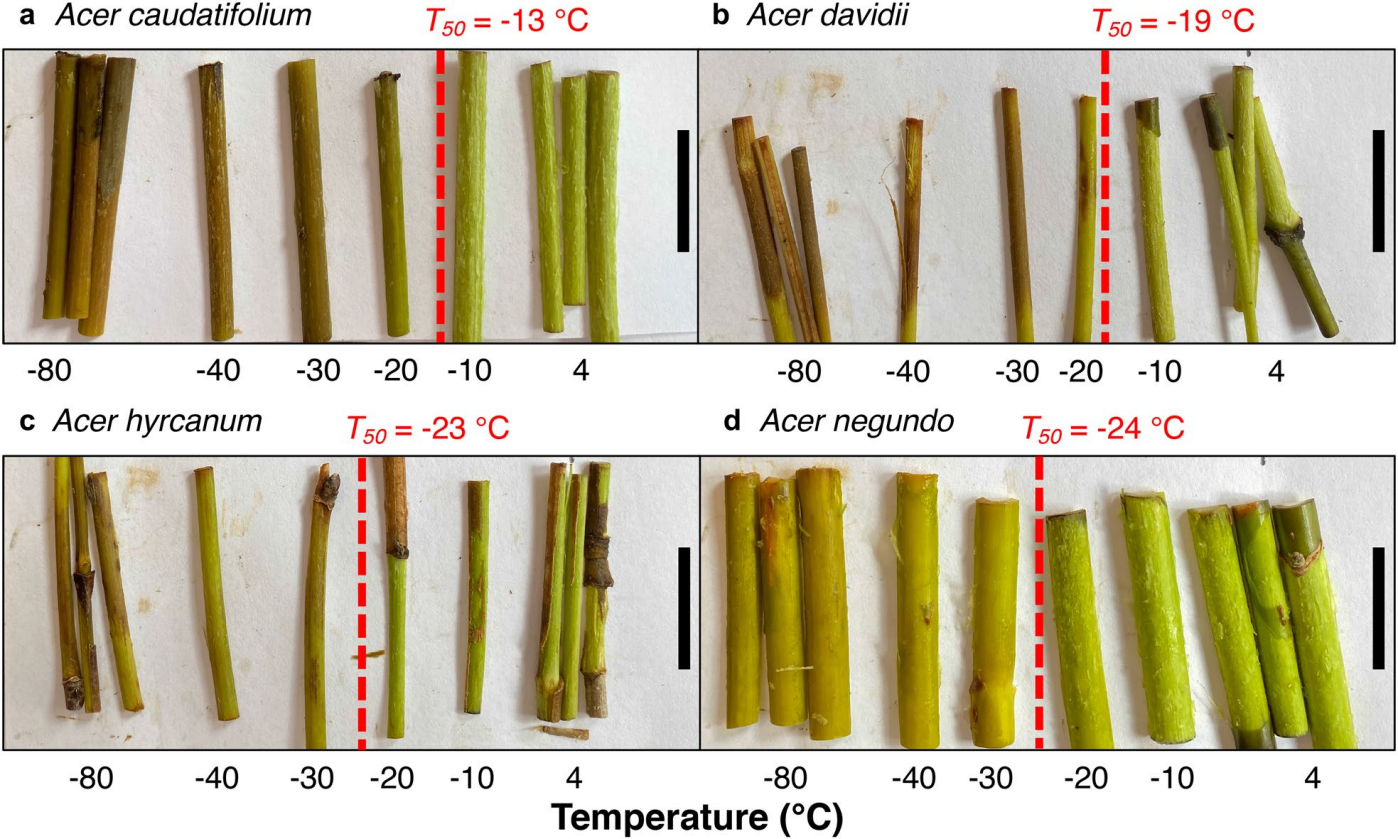
Visual cambial damage corresponded to critical cold hardiness estimated from electrolyte leakage data. Values of T50 given here (Table 1) are calculated using the Lim_logistic_ approach. Representative stem samples following freezing are shown for **A** *Acer caudatifolium*, **B** *A. davidii*, **C** *A. hyrcanum*, and **D** *A. negundo*. Scale bar = 0.5 cm

Yet all four approaches to modeling electrolyte leakage did not perform equally well in predicting critical temperatures for freezing damage as measured using these other methodologies (Table 2, Additional file 3: Figure S2). Across a range of critical visual damage levels (20, 50, and 80%), *T*_*50*_ extracted from general logistic curves fit using the Lim approach best predicted visual damage (*T*_*50*_ or *T*_*80*_) and species differences in LTEs. Critical values extracted using the Lim approach with Gompertz (rather than general logistic) curve fitting also performed well, but Gompertz curves require secondary calculation from model parameters for *T*_*50*_, and therefore are less intuitive and less frequently used than general logistic curves. For these reasons, in the following analyses, we present electrolyte leakage data analyzed using the Lim_logistic_ protocol: zeroed and stretched data fit to a logistic curve. Critical values for leakage, when determined using this approach, approximate critical values for visually estimated damage despite diversity across species and genotypes (Figs. 3, 4).

**Fig. 5:**
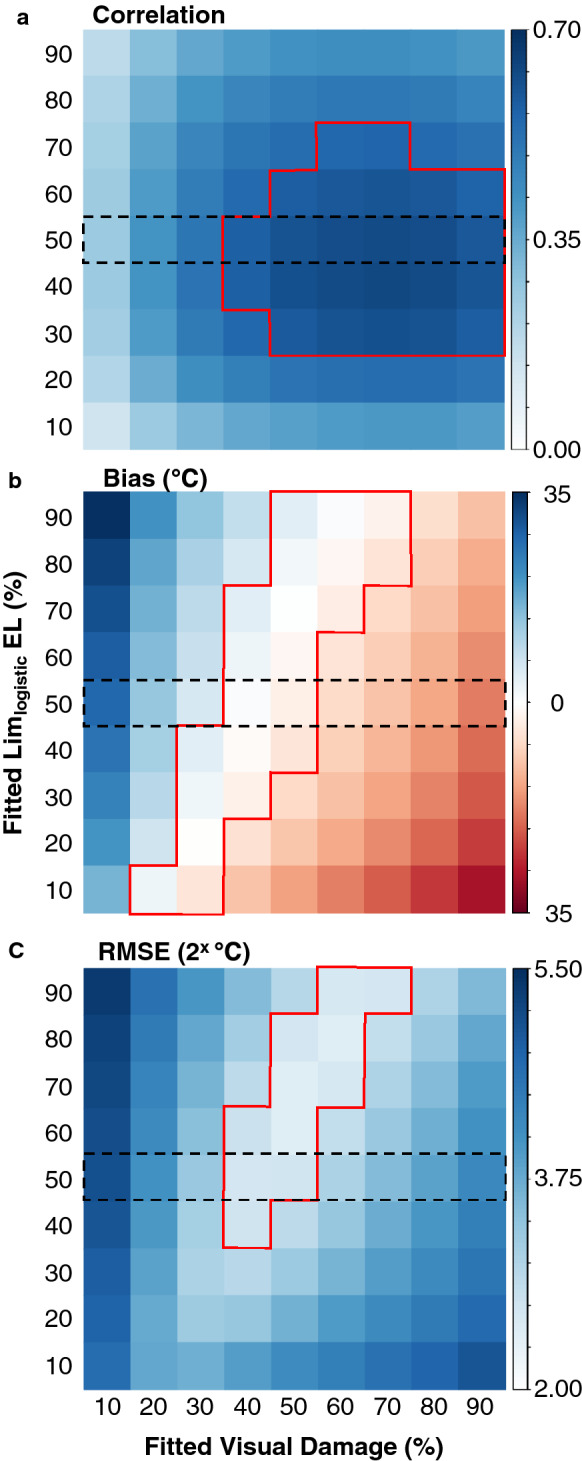
Fitness characteristics of the relationship between fitted values of electrolyte leakage and visual damage. Red contour delimits the area where: **A** Correlation is greater than 0.55; **B** Bias < abs (5 ℃); and **C** RMSE < 7 ℃. Dashed rectangle delimits data used in Additional file 4

We found support for the convention of comparing critical temperatures at the point at which 50% of total leakage or visual damage occurs (Fig. 5, Additional file 4: Figure S3). Critical electrolyte leakage best predicts the temperatures at which between 50 and 80% of visually diagnosed damage occurs, although bias and error between the two assays is lower from 20 to 50% visual damage. Visual damage is accrued at a faster rate than electrolyte leakage (Fig. 3; see values of the *b* parameter in Additional file 2A: Table S1), which explains the minimization of bias and RMSE for their comparison in the 30–60% range of visual damage. Therefore, a comparison of *T*_*50*_ for both electrolyte leakage and visual damage strikes us as an acceptable validation procedure. This comparison indicates that electrolyte leakage explains 36% of the variability in visual damage and underestimates *T*_*50*_ for visual damage by only 3 ℃ (Additional file 4: Figure S3, middle panel).

Species-level LTEs show a different, though consistent, pattern of cold hardiness compared to critical temperatures for either electrolyte leakage or visual damage. Ease of exotherm identification in DTA differed between species, which may account for some of the differences from other methods. Ten of the 12 study species experienced LTEs between − 34.5 and − 38.4 ℃, just above the theoretical supercooling limit for water (roughly – 42 ℃; Table 1A). The other two species, *A. caudatifolium* and *A. davidii*, had very high LTEs, indicating ice formation at roughly – 20 ℃. Because of the bimodal distribution of species means in LTEs, there was not a significant relationship between this critical value and *T*_*50*_ for electrolyte leakage (⍴ = 0.45, p = 0.41) or *T*_*50*_ for visual damage (⍴ = 0.31, p = 0.64).

### Data collected using a novel liquid nitrogen control commensurate with those collected using an autoclaving control

**Fig. 6:**
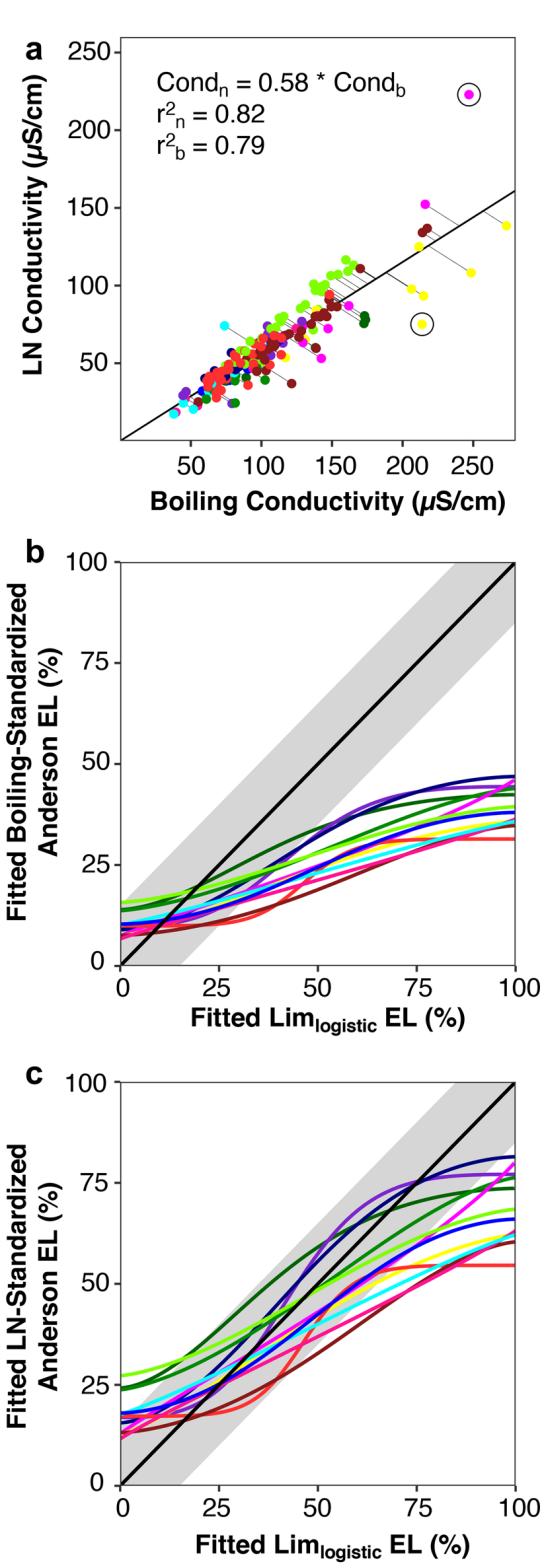
**A** Sample conductivity following boiling predicts conductivity following immersion in liquid nitrogen across a range of values and for diverse species (color-coding indicates species as in Fig. 2). Circled points are statistical outliers and lines indicate Deming regression error. r^2^ were calculated based on residuals in each direction. **B** When a boiling standard is used, electrolyte leakage values derived using different curve-fitting procedures (e.g. Anderson vs. Lim_logistic_) are not comparable above ~ 25% leakage. **C** However, use of a liquid nitrogen standard makes outputs of these two routines more comparable. Grey bar indicates a range of values within 15% of the 1:1 line

Electrolyte leakage induced by immersion in liquid nitrogen generated an average of 58% of the leakage induced by the standard autoclaving approach (an autoclave cycle at 120 ℃) and an average of 184% of the leakage induced by freezing at – 80 ℃. Deming regression of nitrogen-induced leakage based on autoclaving-induced leakage with a zero intercept yields an allometric equation of

$${Conductivity}_{Liquid N}= 0.576\times {Conductivity}_{Autoclaving}$$

with two outliers removed and a 95% confidence interval around the slope ranging from 0.557 to 0.595 (Fig. 6). Increased variance made this relationship weaker for physically larger samples (in this case, those with wider diameter), which leaked more electrolytes and had higher conductivity.

### Use of a liquid nitrogen control provides more consistent estimates of cold hardiness across different approaches to modeling leakage data

Allometric adjustment of leakage values (*R* or *I*) in which an autoclaving standard was used to estimate damage that would have resulted from use of a liquid nitrogen standard led to a considerable improvement in the consistency among approaches for converting electrolyte leakage data into estimates of cold hardiness. For instance, when an autoclaving standard is used, leakage estimated using the Anderson and the Lim_logistic_ approach are not commensurate above very low levels of damage (Fig. 6B, Additional file 5: Figure S4)—with overall bias at 51% and RMSE = 55%. When leakage values are adjusted using the allometric equation presented above to assume a liquid nitrogen standard, leakage estimated using the Anderson approach increases, creating greater concordance between the two approaches (Fig. 6C)—bias is on average 25% and RMSE reaches 29%. If the two approaches are evaluated in a narrower range, between 25 and 75% of EL based on the Lim_logistic_ approach, bias drops to 6% for the liquid nitrogen standard vs 26% for autoclaving, and RMSE to 11 and 28% for liquid nitrogen and autoclaving, respectively.

### Re-analysis of previously published data suggests overestimation of cold hardiness

Re-analysis of raw electrolyte leakage data from [11] underscores the consequences of variation in the major approaches to analyzing data generated using this method. Each of the four approaches we used returned higher critical temperatures—indicating less cold hardiness—for the oak study system than those reported originally by these authors (Table 1). For instance, using the Lim_logistic_ approach, we estimated that unacclimated woody samples accrued 50% freezing damage between − 12° and – 15 ℃; originally published estimates ranged from − 13° and – 20 ℃. Our estimated range of critical values for acclimated samples (− 21° and – 25 ℃) was also considerably warmer than the originally reported one (− 29° and – 34 ℃).

We also observed that, even though Fallon and Cavender-Bares only froze oak woody tissues to a low temperature of – 40 ℃, doing so appeared to cause levels of electrolyte leakage approaching or even exceeding those instigated by autoclaving (Additional file 2B: Table S2). This contrasts with our findings, in which liquid nitrogen immersion (− 200 ℃) only generated about 58% of the leakage caused by autoclaving woody tissue from maples. Our figure of 58% maximum leakage comports with those from our re-analysis of Kreyling and colleagues’ [10] work, in which the Anderson approach was employed and both surveyed maple species showed similar levels of maximum leakage (58–68% in March; Additional file 2B: Table S2).

Indeed, our extrapolation of findings from Kreyling and colleagues’ [10] work suggests that patterns of maximum freezing-induced leakage likely vary among species and over time (Additional file 2B: Table S2). Notably, we observe that some genera, such as the redwoods (subfamily Sequoioideae) and beeches (*Fagus* spp.), appear to release virtually all of their intracellular electrolytes when immersed in liquid nitrogen (− 200 ℃), while others, such as the maples and pines (*Pinus* spp.), resist leakage, even when unacclimated to freezing. We also note a phenological pattern in maximum leakage, which, per Kreyling and colleagues’ findings, was highest in autumn (November), and lowest at the end of winter (February), before increasing again with springtime deacclimation (March; Additional file 2B: Table S2). This pattern is also observed in some oak species in maximum leakage in [11], where samples collected in the summer had generally higher maximum leakage than in the winter—though the lowest temperature used was − 40 ℃. This phenological signal in maximum leakage suggests that the electrolyte leakage at a single temperature cannot be used to compare even the same genotype across time when using autoclaving as a control [36]—but may be possible with a liquid nitrogen control. Differences in maximum leakage among clades also suggest that further research could be performed to understand what portion of electrolytes remains insoluble during freezing at liquid nitrogen temperatures but becomes soluble through boiling or autoclaving.

### Neither use of short incubation times nor choice of boiling, autoclaving, or liquid nitrogen immersion impairs estimates of cold hardiness

Though it was not the main focus of our experiment, we assessed the sensitivity of our electrolyte leakage measurements to variation in the incubation time elapsing between freezing treatments and maximum leakage control, with three different controls: boiling (100 ℃), autoclaving (120 ℃) or liquid nitrogen immersion. We found that longer incubation times following experimental freezing (down to – 80 ℃) led to higher sample conductivity, but that there was no relationship between incubation time and conductivity following either autoclaving or liquid nitrogen immersion (Additional files 3, 6). In particular, conductivity measured after one to two days of incubation following experimental freezing was statistically indistinguishable, while conductivity continued to increase significantly on days five and seven. On the other hand, post-autoclaving or post-liquid nitrogen conductivity did not show a significant, linear relationship with incubation time.

Estimates of *T*_*50*_ derived from samples incubated for five days were generally within a few degrees of those associated with shorter incubations, but, by five days, species-level differences in cold hardiness were no longer reflected in *T*_*50*_ (Additional files 2D, 6). Estimates of cold hardiness from samples incubated for seven days became increasingly variable across methods, potentially reflecting microbial growth on samples (Additional file 7). As such, we conclude that measuring leakage for up to two days following experimental freezing does not substantially bias estimates of cold hardiness, while waiting longer for leakage measurements (up to a week in our comparisons) may actually produce biased or unreliable results.

## Discussion

We compared late-winter cold hardiness of woody tissue in 12 maple species using our updated electrolyte leakage protocol and validated these measurements against other metrics of cold hardiness. We found general agreement among critical values from four distinct approaches to extracting critical cold hardiness values from electrolyte leakage data, as a result of using relative parameters of fitted curves rather than leakage in absolute terms (i.e., values of *R*). However, we recommend the use of critical values associated with 50% damage obtained using the Lim_logistic_ approach. These critical temperatures correspond well across genotype and species with the temperature at which 50% of twig tissue had experienced cambial browning as determined visually. Finally, we found that liquid nitrogen immersion as a control produces electrolyte leakage data commensurate with that produced with an autoclaving control, but can be applied more consistently and is better suited to samples with low levels of maximum freezing-induced leakage.

### Electrolyte leakage data should be adjusted to account for minimum and maximum damage

In surveying the existing electrolyte leakage literature and developing this protocol, we noted consequential variability in the way that raw leakage data are converted to critical values of cold hardiness. As a result, it is possible to compare cold hardiness among treatment groups or species within a single study, but particular values of *R* or *I* may not be transferable across research groups or study systems. We classified the diverse published protocols into four approaches (Fig. 1) based on whether or not authors standardize raw data based on minimally damaged (unfrozen) or deep-frozen (maximally damaged) controls and on what class of curve is fit to adjusted data (general logistic or Gompertz).

Of the four resultant classes of protocols, we recommend the use of either of the Lim approaches (Lim_logistic_ or Lim_gompertz_), in which all leakage data are converted to ${I}_{T,adj}$ through zeroing against leakage from non-freezing (e.g., 4 ℃) and stretching against leakage from deep-freezing (e.g., − 80 ℃) treatments. Though we chose to fit leakage data to a general logistic curve (as in [20]) for reasons of parsimony and ease of interpretation, fitting to a Gompertz curve [13, 22–24] generates similar results. Critical values obtained from this approach best predicted visually observed cambial browning across a variety of damage thresholds (20–80%; Additional file 3), including critical 50% values (Fig. 5) across genotypes and species. (However, we do note that visual browning occurred across a smaller range of temperatures [Table 1]). The values of *R* and *I* we observed also demonstrate the need for the correction of *R* or *I* into *I*_*T,adj*_ (i.e., using relativized measurements in the curve estimates): although in *R* and *I*, an absolute value of 50% damage was not reached, a clear plateau is observed in leakage values below − 40 ℃ (Fig. 2). The use of absolute 50% damage (*R* = 50%) as the metric for cold hardiness level would have resulted in all species having cold hardiness > − 80 ℃ in our study, an unrealistic outcome. This occurred despite the fact that we did not use a controlled, slow thawing, which may have resulted in infliction of additional stress on our samples [36].

Adoption of the approach we recommend will likely affect reporting of estimates of cold hardiness derived from electrolyte leakage data. For instance, we found that critical temperatures (LT_50_) for our maple samples estimated using the Lim_logistic_ approach were highly correlated with (*R*^2^ = 0.81), but not identical to, estimates generated through other approaches (Tables 1, 2). And application of this approach in our re-analysis of Fallon and Cavender-Bares’ [11] findings produced warmer (less cold hardy) estimates of LT_50_ by 1–9 ℃ across species and degree of cold acclimation. We attribute this pattern to Fallon and Cavender-Bares’ [11] use of absolute *I* values for estimation of 50% damage (Fig. 1B). However, as discussed previously, plateaus in their data indicate maximum damage may not occur at 100% of heat killing leakage. In fact, even at liquid nitrogen temperatures, leakage values can fall far below 100%, and even below 30% (Additional file 2B: Table S2), spuriously suggesting that tissue is hardy to temperatures below − 196 ℃.

### Electrolyte leakage corresponds to visual damage, but not low temperature exotherms

To further explore the biological meaningfulness of electrolyte leakage measurements, we validated critical values from the Lim_logistic_ approach against not only critical values of visual damage, but also LTEs corresponding to the formation of intracellular ice. We found a generally high degree of correlation between damage measured by both leakage and browning (Additional file 3: Figure S2), especially when leakage was between 50 and 80% of its observed maximum (Additional file 4: Figure S3). For instance, across the 12 measured maple species, LT_50_ (calculated using the Lim_logistic_ approach) was highly and significantly correlated with browning (⍴ = 0.58, n = 36, p < 0.001; Table 2). Furthermore, the ranges of these critical values also mostly overlapped, with critical damage occurring between − 13° and – 32 ℃ (Table 1). As such, we contribute to the evidence that cellular damage measured via electrolyte leakage corresponds to visually apparent damage to plant cambial tissue in response to freezing [8, 10, 13, 16, 17, 22, 37, 38].

On the other hand, we found that critical electrolyte leakage generally occurred at warmer temperatures than did the formation of symplastic ice (the LTE). We documented relatively uniform LTEs ranging from − 35° to – 38 ℃ for ten species, indicating that these trees could still prevent ice formation in their cells at temperatures far below those they might ever experience in their native ranges. The other two species, *A. caudatifolium* and *A. davidii*, both adapted to relatively warm East Asian climates, had high LTEs (− 20 ℃). Even in these cases, LTEs occurred at temperatures below critical leakage temperatures, suggesting that the two methods capture different aspects of cold hardiness. This finding is consistent with past documentation that LTEs can diverge from critical values for leakage during the growing season but converge when plants are fully cold-acclimated in the winter months [13, 27]. As such, we find evidence that visual damage, but not LTEs, should serve as a useful validation of cold hardiness methods made with electrolyte leakage.

### Liquid nitrogen as a negative control can enhance the use of the electrolyte leakage approach

We found that electrolyte leakage values derived from an autoclaved control and a liquid nitrogen immersion control are highly correlated (R^2^ ~ 80%; Fig. 6A). Because freezing samples at roughly – 200 ℃ by liquid nitrogen immersion is less damaging to woody tissue than autoclaving, raw leakage values calculated using our new control are roughly 58% of the values calculated using an autoclaving control. Given all this, and for the sake of transferability of findings, we conclude that values of *R* or *I* calculated using either method can be compared across studies using the linear equation given in Fig. 6. But, since leakage data produced using either method are linearly related, does the choice of heat killing versus liquid nitrogen immersion even matter? Based on our findings, we argue that use of liquid nitrogen bestows four advantages.

First, as surveyed above, there are a wide variety of heat killing protocols used in electrolyte leakage studies; variability exists in both temperature and intended duration of heat killing, likely hindering generalization of findings. Furthermore, duration of heat killing treatments is often not even obvious: it is unclear whether duration of actual exposure to a target temperature or total time in hot water bath is reported (e.g., “killed in a boiling water bath for 20 min” [39]) or an autoclave cycle (which usually also includes higher pressure to prevent boiling over). Liquid nitrogen immersion, on the other hand, exposes samples to a physically constrained range of temperatures (− 196 to – 210 ℃) for a much more obvious duration of time (the time of immersion).

Second, a liquid nitrogen control may be more suitable for samples collected from cold-hardy or highly acclimated plants or for any sample with low levels of maximum freezing-induced leakage. Because use of an autoclaving standard damages samples more than immersion in liquid nitrogen (Fig. 6A), the former approach produces lower values of *I* or *R* across freezing temperatures. As a result, apparent freezing-induced leakage measured using an autoclaving control often remains low, failing to reach 50% of maximum damage. Low values of *R* or *I*, in turn, are often less commensurate across different approaches to calculating cold hardiness using electrolyte leakage data. For instance, in our comparison of leakage values from the same raw data but calculated using the Anderson vs. Lim_logistic_ approaches (Fig. 6B, C), the two approaches are only commensurate at low damage levels when data are relativized against autoclaving. On the other hand, when data are relativized against liquid nitrogen immersion, the two approaches indicate relatively similar damage across a wider range of temperatures, especially for cold-hardy samples like our acclimated maples or the maples and pines in Kreyling and colleagues’ [10] study (Additional files 2E, 5), or the acclimated boreal conifers in Strimbeck and colleagues’ experiments [14]. Therefore, use of a liquid nitrogen control or conversion of *R* or *I* to assume a liquid nitrogen control facilitates comparison and synthesis across diverse approaches to measuring cold hardiness using electrolyte leakage. A caveat to this argument is our finding, through re-analysis of Fallon and Cavender-Bares’ [11] study of southwestern U.S. oaks, that, when maximum freezing-induced leakage is high, a heat killing control can be sufficient for estimation of cold hardiness using electrolyte leakage. Additionally, some species in specific freezing conditions are able to withstand immersion in liquid nitrogen [40–44]. Therefore, regardless of the control used, cold hardiness experiments should include some evaluation of survival or damage of tissues to corroborate results found through more objective methods such as electrolyte leakage.

Third, we note that, as expected, liquid nitrogen immersion provides a more realistic proxy of “maximum damage” to samples than did autoclaving. Liquid nitrogen immersion (at roughly – 200 ℃) caused a little less than twice the leakage inflicted by freezing at – 80 ℃, a temperature that none of the world’s woody plants ever experience in a natural context. As such, freezing through liquid nitrogen immersion seems sufficient to inflict the maximum possible damage that could be expected from this mode of injury on woody samples, while avoiding the excessive treatment afforded by heat killing. The choice of heat killing versus liquid nitrogen immersion appears to be less important for non-woody samples (e.g., leaves, crowns [12, 45]), though may still be important for more lignified green tissues such as conifer needles [14, 45].

Fourth and perhaps most obviously, submerging the tubes in liquid nitrogen provides the same physical environment for the samples (ice, compared to boiling liquid water) as they experience when exposed to the damaging stressor of interest. Use of a liquid medium as a control means that the resulting leakage occurring during heat killing may be due to other effects other than only temperature.

## Conclusions

A central recommendation we make for all workers performing electrolyte leakage research is that estimates of cold hardiness using this method should take place across a range of temperatures, and not simply at one freezing temperature of interest. This range must include one temperature above freezing (e.g., 4 ℃) and one or more temperatures chosen to elicit maximum freezing damage such that a plateau in electrolyte leakage values is observed (e.g., <  − 80 ℃ and ideally including submersion in liquid nitrogen). If this approach to sampling is observed, all four of the analytical approaches we surveyed are likely to yield similar critical values of cold hardiness (Tables 1, 2). Generally, we found LT_50_, extracted from data fit to either a general logistic or Gompertz curve following Lim and colleagues’ [13] approach, to best approximate visually observed damage to woody tissue.

We highly recommend the use of the liquid nitrogen control we present here in lieu of boiling or autoclaving. This control will be especially useful for very cold hardy woody samples (or physically small ones) coming from, for instance, acclimated temperate or boreal species prior to leaf-out. And though we offer a simple linear equation for estimation of leakage assuming a liquid nitrogen control from leakage data collected using an autoclaving control (Fig. 6), we suggest some caution in applying this conversion widely, especially outside of the maple genus or to non-woody samples. Application to other systems may benefit from experimental determination of a linear equation relevant to the samples in question.

Finally, we did not expect to find the diversity in maximum freezing-induced electrolyte leakage that we found among species in our own work, in re-analyzed studies (Additional file 2B), and more broadly in the literature [14]. Though this value appears to depend somewhat on the lineage being sampled and on the time of year, it is not clear what underlying mechanism causes such variety in the difference between electrolyte leakage following deep freezing and autoclaving among woody samples. Future work might explore a connection between this maximum freezing-induced leakage and cold hardiness.

## Methods

### Study System

The roughly 120 species of the maple genus (*Acer* L.) are distributed widely throughout the northern hemisphere, having diverged from relatives in the Sapindaceae some 60 million years ago and radiated from their center of diversity in eastern China to mesic environments throughout Asia, Europe, and North America [46]. The maples are a typical temperate, woody genus, including common forest dominants and rare and subordinate species, with species’ native distributions covering areas with mild winters (Taiwan, Mexico) through very cold ones (Scandinavia, the Amur Valley). In the present study, we focus on twelve maple species spanning the maple phylogeny [47, 48] and three principle biogeographic realms colonized by the genus: Asia, North America, and Europe (Additional file 1; [49]).

All plant material was collected from mature, healthy trees at the Arnold Arboretum (42°18′26″ N, 71°07′13 W”, 15–79 m a.s.l) in Boston, MA, USA – an impromptu common garden for cold hardiness studies [14]. The Arboretum has a hot summer continental climate (*cfa* in the Köppen-Geiger system) with a historical mean annual temperature of 9.7 ℃ and mean annual precipitation of 1168 mm. Collections were primarily from accessioned trees in the Arnold’s living collections and occasionally from spontaneous individuals growing on the Arboretum grounds. Information about all sampled trees is provided in Additional file 2F.

We obtained previously published electrolyte leakage data for six southwestern U.S. oak species (*Quercus* spp.; [11]). We also extracted some data from supplements associated with Kreyling and colleagues’ [10] study of diverse woody species at the Ecological Botanical Garden in Bayreuth, Germany.

### Electrolyte leakage measurements

We developed our electrolyte leakage routine by drawing on published accounts and testing a variety of alternative methods. First, we collected material from three genotypes per species (n = 36), completing all sampling on 13 March 2020, noting the phenological stage of each sampled individual. Healthy, one to two year-old stems, typically 3–10 mm in diameter and < 30 cm in length were harvested and placed in sealed plastic bags to prevent desiccation. These were then stored at 4 ℃ to prevent further de/acclimation until sample preparation and testing. Processing consisted of cutting internode stems into 10 mm segments and sealing them in 15 mL plastic test tubes with 2 mL of nanopure water (< 18.2 MΩ). For each genotype, 21 segments were prepared. Allocated across 6 freezing temperatures and a control, these yielded three measurement replicates per genotype. Freezing trials then occurred either 12 or 36 h after sample preparation was completed; samples were randomized across either the first or second day of trials and stored at 4 ℃ prior to freezing.

Freezing trials occurred in a Tenney Environmental (New Columbia, PA, USA) Test Chamber with thermocouple monitoring of temperature using an RDXL4SD data logger (Omega Engineering, Inc., USA). Target temperatures for freezing were: 4 ℃ (controls kept in a growth chamber); − 10, − 20, − 30, − 40, and − 60 °C (in the Tenney Chamber); and − 80 °C (in a separate freezer). Samples in the Tenney Chamber were initially cooled from a storage temperature of 4 to 0 ℃ over thirty minutes and then held at 0 ℃ for an additional thirty minutes. They were then stepped down to − 10 ℃ at a rate of − 0.33 °C/min and held for one hour at the target temperature. This routine was repeated for − 20, − 30, − 40, and − 60 °C with the rate of temperature change between − 40 and − 60 °C twice that of the rate between other steps. After one hour at − 60 °C, samples assigned to a − 80 °C target were moved to a separate deep freezer and held there for one to two hours. Samples were removed from the Tenney Chamber or deep freezer and allowed to return to room temperature on the benchtop after being held at their target temperature. Visual inspection showed that water in all tubes froze completely without exogenous ice seeding or inclusion of steel shot to promote ice formation within the first freezing step (− 10 ℃). Thermocouple readings indicate that actual sample temperature was on average 1.5 ℃ colder than the target temperature; we use actual temperature reached (e.g., − 10.5 in lieu of − 10 °C) for analysis.

Following sample thawing, 8 mL of nanopure water were added to each test tube and tubes were shaken at 100 rpm and room temperature for 16 h. Conductivity of solution in each tube was then read with a conductivity probe calibrated with a low-concentration (0–200 μS/cm) standard (Vernier Software and Technology, Beaverton, OR, USA). Samples were hand-shaken immediately before conductivity measurements; we found that this was essential for accurate readings.

### A novel control: Liquid nitrogen immersion

Out of concern with the realism and consistency of the current set of methods used in electrolyte leakage studies, we have developed and employed here an alternative approach, immersing all sample tubes in liquid nitrogen (at least − 200 ℃ as indicated by thermocouple readings) for thirty minutes following initial conductivity readings. Samples were then allowed to thaw on the bench and shaken at room temperature and 100 rpm for 16 h. Final conductivity measurements were then collected as described above.

To validate the use of this new control, a separate set of stem samples (n = 176) from all 12 study species collected on 3 and 10 February 2020 was used to carry out the protocol described above. Following freezing in liquid nitrogen, we measured their conductivity, then autoclaved them at 120 ℃ for thirty minutes, loosening tube caps prior to autoclaving to reduce evaporation while preventing overpressurization of tubes. (We autoclaved them at high heat to maximize electrolyte leakage.) We then agitated tubes for 16 h and measured conductivity, both as described above, allowing for comparison between leakage following liquid nitrogen immersion and autoclaving.

Previous studies have suggested that frozen samples may not release all diffusible electrolytes for up to a week following freezing and that samples exposed to autoclaving for short periods of time (e.g., 15 min.) will continue to leak electrolytes for ten days or more [16]. In order to ascertain that our incubation timing (both post-freezing and post-autoclaving or liquid nitrogen immersion) and autoclave intensities were sufficient to capture electrolyte diffusion, we carried out an additional experiment on only two species, *A. caudatifolium* and *A. campestre*. These two maples represented the two extremes of cold hardiness in our main experiments (Table 1), occupy distinct habitats (Additional file 1), and belong to phylogenetically distinct sections [48]. For this additional experiment, we collected stem sections of one genotype of each species (with three measurement replicates per genotype collected) on 29 December 2020 and measured using the same electrolyte leakage protocol described above while varying the amount of time samples incubated after temperature treatment, and after maximum damage controls (boiling, autoclaving, and liquid nitrogen). As such, conductivity of stem segments of both species was measured 1, 2, 5, or 7 days post-freezing and then exposed to either boiling at 100 ℃ in the Tenney Chamber, autoclaving at 120 ℃, or liquid nitrogen immersion (N = 504). Segments were then allowed to incubate for the same length of time as their post-freezing incubation and conductivity was re-measured. This experiment crossed incubation time (one day through one week) with control method (boiling, autoclaving, or liquid nitrogen) for two species expected to vary in their cold hardiness, producing 24 factor-level combinations.

### Validation of electrolyte leakage

To validate estimates of cold hardiness from electrolyte leakage, we performed two additional cold hardiness assays on stem tissue from sampled individuals: differential thermal analysis and visual inspection of freezing damage. We briefly describe these procedures below.

#### Differential thermal analysis

Differential thermal analysis (DTAs; [50–52]) involves cooling plant tissue of interest to progressively lower temperatures while using thermoelectric sensors to detect the tissue’s release of heat associated with the freezing of water, an exothermic reaction. Extracellular water in stems, buds, etc. freezes at relatively warm sub-zero temperatures, producing a release of heat or high-temperature exotherm (HTE); the freezing of this extracellular water is not considered harmful to acclimated, cold-adapted woody plants. Intracellular water, on the other hand, will generally supercool to much lower temperatures, reaching a limit at about − 42 ℃. Freezing of this supercooled water, which is associated with catastrophic damage to affected cells, results in a low-temperature exotherm (LTE), which can be detected through DTA and compared to results from electrolyte leakage assays [8, 17, 19, 27].

For DTA measurements, carried out on 16 March 2020, we used the same material collected for our electrolyte leakage trials; this material had been stored with proximal ends inserted in water, at 2 ℃ for the intervening time (3 days). For each genotype (n = 36), we cut ten 30 mm segments of the same internodal stem tissue and pooled these measurement replicates into a cell in which they were exposed to thermoelectric modules, which can detect exotherms and convert their thermal signals to voltage. Samples were then cooled at a rate of − 4 ℃/hour to − 60 ℃. Exotherm-associated voltages were collected using a Keithley Multimeter Data Acquisition System (Keithley Instruments, Cleveland, OH, USA). HTE peaks were discarded and LTE peaks were manually curated in Microsoft Excel prior to statistical analysis.

#### Visual damage inspection

We also visually inspected damage to cortical tissues cells following freezing; this metric serves as an intuitive and holistic, though labor-intensive method for assessing cold hardiness [8, 10, 17, 22, 53]. On 16 March 2020, we prepared samples for freezing followed by visual damage inspection from the same material used in other trials. For each genotype (n = 36), and each temperature treatment, we cut three 30 mm segments of internodal stem tissue and incubated them in plastic scintillation vials with 2 mL water at 2 ℃ overnight. On 17 March, we froze these vials using the same freezing routine as the one employed in our electrolyte leakage measurements. One set of vials was kept at 4 ℃. The others were frozen to − 10, − 20, − 30, − 40, and − 80 °C. After removal from the freezer, vials were allowed to thaw at 4 ℃ for 24 h, then incubated with ends in water, at room temperature for six days. At this time, they were evaluated using a quartile (0, 25, 50, 75 or 100% brown) damage scale. An initial plan included image analysis to determine damage levels (e.g., [14]), but COVID-19- related restrictions prevented laboratory and equipment access that would allow proper imaging.

### Data analysis

All analyses were carried out in R (ver. 3.6.3, [54]).

#### Treatment of raw electrolyte leakage data

Raw electrolyte leakage at each temperature was normalized into a relative electrolyte leakage (*R*_*T*_) at temperature *T*:

$$R_{T} = L_{T} /L_{k}$$

where $L_{T}$ is the conductivity measured from a sample frozen at *T* and $L_{k}$ is the conductivity of the same sample after autoclaving. *R*_*T*_ is the measurement used for damage estimation based on the Anderson et al. [12] approach. We then converted *R*_*T*_ to Index of Injury ($I_{T}$) for any given temperature *T* following Flint and colleagues’ [7] method such that:

$$I_{T} = 100 \times \frac{{\left( {R_{T} - R_{o} } \right)}}{{\left( {1 - R_{o} } \right)}}$$

where $R_{o}$ = *R*_*T*_ for an unfrozen control. In our case, the unfrozen control was kept at 4 ℃. Therefore, *I*_*T*_ = 0 at *T* = 4 ℃.

The third method employed was a modified version of Lim and colleagues’ [13]. This adjustment builds upon the apparent plateau observed in levels of damage in lower temperatures and assumes maximum damage occurred within the temperatures tested:

$$I_{T, adj} = 100 \times I_{T} /I_{max} \equiv 100 \times \frac{{\left( {R_{T} - R_{o} } \right)}}{{\left( {R_{max} - R_{o} } \right)}}$$

where $I_{max}$ and *R*_*max*_ are the maximum unadjusted injury and relative conductivity measured for any genotype (usually attained at either − 60 or − 80 ℃).

#### Analysis of electrolyte leakage and visual damage data

Electrolyte leakage values of *R*_*T*_, *I*_*T*_, and *I*_*T, adj*_, and visual damage (*VD*) were then modeled for each genotype using a four-parameter log-logistic model:

$$R_{T} , I_{T} , I_{adj,T} , VD_{T} = c + \frac{d - c}{{1 + e^{{\left( {b \times \left( {\left( T \right) - \left( u \right) } \right)} \right)}} }}$$

where *T* is the temperature, *e* is euler’s number, and *d*, *c, b*, and *u* are the parameters estimated: *c* is the lower limit (*c* = 0 for *I*_*T*_, *I*_*adj, T*_, and *VD*_*T*_); *d* is the upper limit (*d* = 100 for *I*_*adj, T*_ and *VD*_*T*_); *b* is the slope associated with the logistic function; and *u* is the inflection point. Here *LT*_*50*_ = *u*. For *I*_*T, adj*_ we also extracted values of *LT*_*20*_ and *LT*_*80*_ for comparison with *LT*_*50.*_ (Additional file 2E: Table S5).

We also modeled *I*_*adj, T*_ using a Gompertz function and extract both *LT*_*50*_* and* $LT_{max}$ as a critical value of cold hardiness as follows:

$$I_{adj,T} = 100 \times e^{{ - b \times e^{ - k \times T} }}$$

$$LT_{max} = \frac{{ - log\left( {1/b} \right)}}{k}$$

where *T* is freezing temperature, *e* is euler’s number, and *b* and *k* are parameters. We fit Gompertz curves for each genotype in our dataset using the *nls* function, whereas log-logistic functions were modeled using the *drm* funditon in the drc package [55].

Critical values of *LT*_*50*_ were extracted from the curves obtained from each genotype for each model for comparison of means at a species level. For *I*_*adj*_, values of *LT*_*20*_ and *LT*_*80*_ were also used. We also calculated the lowest survival temperature (LST) on a species basis for visual damage, which we defined as the lowest temperature at which no stem segment experienced more than 50% damage [8, 50]. Species differences for these were analyzed using simple linear regression models and Type I ANOVAs. Tukey’s HSD post-hoc test (*HSD.test*, “agricolae”; [56]) is used to discriminate among groups in cases of significant difference.

Bivariate correlation tests were used to compare values of *LT*_*20*_, *LT*_*50*_ and *LT*_*80*_ for the three electrolyte leakage models with *LT*_*20*_, *LT*_*50*_ and *LT*_*80*_ and LST values for visual damage. Further comparisons in terms of correlation, bias, and RMSE were made from *LT*_*10*_ to *LT*_*90*_ in 10% steps for *I*_*adj*_ and *VD*.

Using raw electrolyte leakage data from a different study comparing acclimated and unacclimated oaks [11], we followed the same protocol described to obtain *R*, *I* and *I*_*adj*_. Since there were multiple genotypes within each species, but no repetition within temperatures for each genotype, the control level used for *I* and *I*_*adj*_ was the lowest electrolyte leakage at either 4 ℃ or − 5 ℃ within each genotype. The three forms of leakage data were used for log-logistic curve estimations of *T*_*50*_, whereas *I*_*adj*_ was used for *T*_*50*_ and *T*_*max*_ estimations based on the Gompertz function as well.

#### Comparison of liquid nitrogen and autoclaving leakage

To compare the values of electrolyte leakage in both types of control, Deming (or “least rectangle”) regressions were fit. This type of regression takes into account errors in both axes to find the best fit line. Although we did not include a pure water sample control in the measurements, initial fitting showed a non-significant intercept, and therefore further fitting was done with a zero-intercept model. Regular least-squares R^2^ values were calculated in both directions to evaluate fitness of the model, from which a linear allometric relationship was extracted.

Damage estimated based on electrolyte leakage standardized on the liquid nitrogen control was then calculated and fit based on *R* and *I* approaches. Fitted values were then compared between values of *I*_*adj*_—for which the control method has no influence—and *R* and *I* for both autoclaving and liquid nitrogen control.

To understand how leakage following heat killing compares to that caused by deep freezing across study systems, we compared our maple data to leakage measurements taken on diverse species in two other studies. We extracted additional *R* data at liquid nitrogen temperatures from figures in the supplementary data of Kreyling et al. [10] using a grid with 5% increments. This dataset contained electrolyte leakage for 27 species, 3 genotypes per species, at 3 different points: November, February, and March. Species and time differences were analyzed using simple linear regression models and Type I ANOVAs. Tukey’s HSD post-hoc tests were used to discriminate among groups. We also used data from the lowest temperature used by Fallon and Cavender-Bares [11] for similar analyses using species and time (acclimated vs. unacclimated) as variables. Although the Deming regressions used in our data are more appropriate, we used *R* values at liquid nitrogen temperatures and analyzed it using linear regression with species as the explanatory variable for comparison with the other studies.

### Impacts of incubation time and control method on electrolyte diffusion

To assess whether electrolyte leakage depended on species identity, control methodology, or incubation time, we built two fixed-effects linear models (using *lm*) of electrolyte leakage measured in our focused study of *A. caudatifolium* and *A. campestre* stem segments. These models both took the form of:

$$L_{T} , L_{K} \sim \beta_{Intercept} + \beta_{Temperature} + \beta_{Species} * \beta_{Incubation} * \beta_{Control} + \varepsilon$$

In this analysis, either leakage following freezing (*L*_*T*_) or exposure to autoclaving or liquid nitrogen (*L*_*K*_) was modeled as a linear function of species identity, incubation time (a continuous variable consisting of 1, 2, 5, or 7 days), and control type (autoclaving at 100 ℃, autoclaving at 120 ℃, or liquid nitrogen immersion). Temperature of freezing treatment (4 to – 80 ℃) was included as a covariate as it is expected to affect sample conductivity. Models were fit with all interactions except those with freezing temperature and analyzed using a conservative Type III ANOVA. Differences among factor-level combinations were assessed using Tukey tests, as described above. For further comparison of the impacts of differences in incubation time and control type on estimates of cold hardiness, critical values (*T*_*50*_) were extracted for each factor-level combination using the Lim_logistic_ approach.

## Supplementary Information

**Additional file 1**: **Figure S1**. A) Native and naturalized distributions of the 12 maple study species, which are distributed across the North American (red/pink), European (green/yellow), and Asian (blue/purple) extent of the genus [49]. Star indicates the location of the Arnold Arboretum. B) Phylogenetic relatedness and ecological descriptions for the study species. Phylogeny and section designations adapted from [47,48].

**Additional file 2**: Tables containing additional information of models tested and data (re-)analysis. **Table S1**. Parameters, error (in parentheses), and pseudo-R2 for models of freezing damage as measured by A-D) electrolyte leakage or E) visual observation of cambial browning. Models are fit to A-C, E) a general logistic curve or D) a Gompertz curve as described in the text. **Table S2**. Maximum freezing-induced leakage (%) attained across several electrolyte leakage studies. Mean separations reflect outcomes of Tukey's HSD post-hoc tests (α = 0.05). A) Data for maple (Acer spp.) species included in the present study. Percentages indicate leakage following immersion in liquid nitrogen divided by leakage following boiling. Superscripts reflect species differences among means. B) Data for oak (Quercus spp.) species from Fallon and Cavender-Bares' [11] study. Percentages indicate leakage following freezing to -40℃ (the lowest freezing temperature used in the study) divided by leakage following boiling. Superscripts reflect differences among means of species-treatment combinations. C) Data for a variety of species assessed during three times of year extracted from supplementary figures from Kreyling et al. [10]. Percentages indicate leakage following immersion in liquid nitrogen divided by leakage following boiling. Two axes of mean separation are presented. Differences among species within the same sampling month are indicated by lowercase letters. Differences among sampling months for a single species are indicated by uppercase letters. Color coding (red to blue) highlights species and temporal patterns in maximum freezing-induced leakage in the Kreyling et al. data. **Table S3**. Results from Type III ANOVA of linear models assessing the consequences of species (A. caudatifolium vs. A. campestre), control type (boiling at 120C, boiling at 125 C, or liquid nitrogen immersion), and incubation time (1, 2, 5, or 7 days post-treatment) on A) conductivity after experimental freezing and B) conductivity after control treatment. Temperature of experimental freezing was included as a covariate.Samples were collected on 29 Dec. 2020. **Table S4**. Critical cold hardiness (T50, degrees C) estimated using the Limlogistic approach for two species, A) Acer caudatifolium and B) A. campestre with control type (boiling at 120C, boiling at 125 C, or liquid nitrogen immersion) and incubation time (1, 2, 5, or 7* days post-treatment) varied. Samples were collected on 29 Dec. 2020. **Table S5**. Species means of critical temperatures at which tissue accrued 20% and 80% damage as measured with electrolyte leakage and calculated using the Limlogistic approach. In cases for which differences among species are significant, superscripts indicate results of a Tukey's HSD post-hoc test (α = 0.05). **Table S6**. Description of all sampled plants. Genotypes of 10 species are all accessioned individuals in the Arnold Arboretum's living collections, as detailed below. Samples of A. negundo were collected from three spontaneously occurring plants found in the Arboretum's Bussey Brook Meadow. Samples of A. platanoides were similarly collected from three spontaneous individuals growing on the Arboretum's Weld Hill parcel.

**Additional file 3**: **Figure S2**. A) Validation of critical values from four approaches to modeling electrolyte leakage (as in Fig. 1) against visual estimates of freezing damage. Critical values reflect either 20%, 50%, or 80% electrolyte leakage (rows) or visual damage (columns). The rightmost column indicates lowest survival temperature (LST), the lowest temperature at which stems experienced < 50% damage. Pie wedge size indicates correlation. B) 50% electrolyte leakage values using the Limlogistic approach (orange box) best predicted visual damage in the 40-60% damage range.

**Additional file 4**: **Figure S3**. Critical electrolyte leakage (estimated using the Limlogistic approach) best approximates 50% visual damage when leakage is between 50 and 80%. Bias, though, is lowest from 20 to 50% leakage. Color-coding indicates species (see bottom left panel). Error reflects variation among genotypes of a given species.

**Additional file 5**: **Figure S4**. When a boiling standard is used, electrolyte leakage values derived using different curve-fitting procedures (e.g. Anderson vs. Limlogistic vs. Flint approaches) are not comparable above ~25% leakage (A vs. B). However, use of a liquid nitrogen standard makes outputs of these two routines more comparable (C vs. D). Grey bar indicates a range of values within 15% of the 1:1 line.

**Additional file 6**: **Figure S5**. A) Electrolyte leakage increased gradually over seven days following experimental freezing (light blue), but not exposure to a boiling or liquid nitrogen control (turquoise), with no evidence of a significant difference when conductivity was measured over the first 48 hours after freezing. Lowercase letters indicate significant differences in conductivity measured at different time points at the 0.05 level based on models reported in Additional file 2C panels A (light blue, a-c) and B (turquoise, d-e). B) As a result of this pattern, estimates of critical values for cold hardiness (T50) are consistent and reflect species differences when samples were incubated for one or two days, but not when they were incubated for 5 or 7 days (Additional file 2D).

**Additional file 7**: **Figure S6**. Stem segments incubated for longer than five days following control treatment (boiling or liquid nitrogen immersion) tended to deteriorate, showing evidence of microbial growth.

## Data Availability

All data generated by the authors during this study (on *Acer* samples) are included in this published article and its supplementary information files. Re-analyzed data provided from other authors are available from the corresponding author on reasonable request and pending permission from the originating author(s). Code and maple data are also available at https://github.com/apkovaleski/EL_Methods.

## References

[1] Gusta LV, Wisniewski M (2013). Understanding plant cold hardiness: An opinion. Physiol Plant.

[2] Edwards EJ, Chatelet DS, Chen BC, Ong JY, Tagane S, Kanemitsu H (2017). Convergence, consilience, and the evolution of temperate deciduous forests. Am Nat.

[3] Koehler K, Center A, Cavender-Bares J (2012). Evidence for a freezing tolerance-growth rate trade-off in the live oaks (Quercus series Virentes) across the tropical-temperate divide. New Phytol.

[4] Hawkins BA, Rueda M, Rangel TF, Field R, Diniz-Filho JAF (2014). Community phylogenetics at the biogeographical scale: Cold tolerance, niche conservatism and the structure of North American forests. J Biogeogr.

[5] Muffler L, Beierkuhnlein C, Aas G, Jentsch A, Schweiger AH, Zohner C (2016). Distribution ranges and spring phenology explain late frost sensitivity in 170 woody plants from the Northern Hemisphere. Glob Ecol Biogeogr.

[6] Dexter ST, Tottingham WE, Graber LF (1932). Investigations of the hardiness of plants by measurement of electrical conductivity. Plant Physiol.

[7] Flint HL, Boyce BR, Beattie DJ (1967). Index of injury—a useful expression of freezing injury to plant tissues as determined by the electrolytic method. Can J Plant Sci.

[8] Aniśko T, Lindstrom OM (1995). Applying the Richards function in freezing tolerance determination with electrolyte and phenolic leakage techniques. Physiol Plant.

[9] Lenz A, Hoch G, Vitasse Y, Körner C (2013). European deciduous trees exhibit similar safety margins against damage by spring freeze events along elevational gradients. New Phytol.

[10] Kreyling J, Schmid S, Aas G (2015). Cold tolerance of tree species is related to the climate of their native ranges. J Biogeogr.

[11] Fallon B, Cavender-Bares J (2018). Leaf-level trade-offs between drought avoidance and desiccation recovery drive elevation stratification in arid oaks. Ecosphere [Internet]..

[12] Anderson JA, Kenna MP, Taliaferro CM (1988). Cold hardiness of ‘Midiron’ and ‘Tifgreen’ Bermudagrass. HortScience.

[13] Lim CC, Arora R, Townsend EC (1998). Comparing Gompertz and Richards Functions to estimate freezing injury in Rhododendron using electrolyte leakage. J Am Soc Hortic Sci.

[14] Strimbeck GR, Kjellsen TD, Schaberg PG, Murakami PF (2007). Cold in the common garden: comparative low-temperature tolerance of boreal and temperate conifer foliage. Trees.

[15] Strimbeck GR, Schaberg PG, DeHayes DH, Shane JB, Hawley GJ (1995). Midwinter dehardening of montane red spruce during a natural thaw. Can J For Res.

[16] Deans JD, Billington HL, Harvey FJ (1995). Assessment of frost damage to leafless stem tissues of Quercus petraea: a reappraisal of the method of relative conductivity. Forestry.

[17] Fiorino P, Mancuso S (2000). Differential thermal analysis, supercooling and cell viability in organs of Olea europaea at subzero temperatures. Adv Hortic Sci.

[18] Guàrdia M, Charrier G, Vilanova A, Savé R, Ameglio T, Aletà N (2016). Genetics of frost hardiness in Juglans regia L and relationship with growth and phenology. Tree Genet Genomes.

[19] Wu D, Kukkonen S, Luoranen J, Pulkkinen P, Heinonen J, Pappinen A (2019). Influence of late autumn preconditioning temperature on frost hardiness of apple blueberry and blackcurrant saplings. Sci Hortic (Amsterdam).

[20] Ouyang L, Leus L, De Keyser E, Van Labeke MC (2019). Seasonal changes in cold hardiness and carbohydrate metabolism in four garden rose cultivars. J Plant Physiol.

[21] Von Fircks HA, Verwijst T (1993). Plant viability as a function of temperature stress: The Richards function applied to data from freezing tests of growing shoots. Plant Physiol.

[22] Lindén L, Palonen P, Lindén M (2000). Relating freeze-induced electrolyte leakage measurements to lethal temperature in red raspberry. J Am Soc Hortic Sci.

[23] Lee JH, Yu DJ, Kim SJ, Choi D, Lee HJ (2012). Intraspecies differences in cold hardiness, carbohydrate content and β-amylase gene expression of Vaccinium corymbosum during cold acclimation and deacclimation. Tree Physiol.

[24] Yu DJ, Hwang JY, Chung SW, Oh HD, Yun SK, Lee HJ (2017). Changes in cold hardiness and carbohydrate content in peach (Prunus persica) trunk bark and wood tissues during cold acclimation and deacclimation. Sci Hortic (Amsterdam).

[25] Stuart NW (1939). Comparative cold hardiness of scion roots from fifty apple varieties. Proc Am Soc Hortic Sci.

[26] Zhang YJ, Bucci SJ, Arias NS, Scholz FG, Hao GY, Cao KF (2016). Freezing resistance in Patagonian woody shrubs: the role of cell wall elasticity and stem vessel size. Tree Physiol.

[27] Arora R, Wisniewski ME, Scorza R (1992). Cold acclimation in genetically related (Sibling) deciduous and evergreen peach (Prunus persica [L] Batsch): I. Seasonal changes in cold hardiness and polypeptides of bark and xylem tissues. Plant Physiol.

[28] Pagter M, Jensen CR, Petersen KK, Liu F, Arora R (2008). Changes in carbohydrates, ABA and bark proteins during seasonal cold acclimation and deacclimation in Hydrangea species differing in cold hardiness. Physiol Plant.

[29] Deacon NJ, Grossman JJ, Cavender-Bares J. Drought and freezing vulnerability of the isolated hybrid aspen Populus x smithii relative to its parental species, P. tremuloides and P. grandidentata. Ecol Evol. 2019; 9:8062–8074. 10.1002/ece3.5364 PMC6662423 31380071

[30] Ouyang L, Leus L, Van Labeke MC (2019). Three-year screening for cold hardiness of garden roses. Sci Hortic (Amsterdam).

[31] Odlum KD, Blake TJ (2008). A comparison of analytical approaches for assessing freezing damage in black spruce using electrolyte leakage methods. Can J Bot.

[32] Repo T, Mononen K, Alvila L, Pakkanen TT, Hänninen H (2008). Cold acclimation of pedunculate oak (Quercus robur L.) at its northernmost distribution range. Environ Exp Bot.

[33] Hodge GR, Dvorak WS, Tighe ME (2012). Comparisons between laboratory and field results of frost tolerance of pines from the southern USA and Mesoamerica planted as exotics. South For.

[34] Cho W, Chandra R, Lee S, Han J, Lee S, Tsetsegmaa G (2020). Cold hardiness of 8 hybrid poplar clones for the introduction to arid and semi-arid areas. Plant Breed Biotechnol.

[35] Zhang MIN, Willison JHM (1987). An improved conductivity method for the measurement of frost hardiness. Can J Bot.

[36] Burr KE, Hawkins CDB, L’Hirondelle SJ, Binder WD, George MF, Repo T, Bigras FJ, Colombo SJ (2001). Methods for measuring cold hardiness of conifers. Conifer cold hardiness.

[37] Palonen P, Lindén L (1999). Dormancy, cold hardiness, dehardening, and rehardening in selected red raspberry cultivars. J Am Soc Hortic Sci.

[38] Savage JA, Cavender-Bares J (2013). Phenological cues drive an apparent trade-off between freezing tolerance and growth in the family Salicaceae. Ecology.

[39] Li H, Li Q, Xing L, Sun G, Zhao X (2020). Comparison of cold hardiness evaluation of woody species by ELLT and TTCLT. HortScience.

[40] Sakai A (1960). Survival of the twig of woody plants at –196 ℃. Nature.

[41] Sakai A (1965). Survival of plant tissue at super-low temperatures III. Relation between effective prefreezing temperatures and the degree of frost hardiness. Plant Physiol.

[42] Sakai A, Yoshida S (1967). Survival of plant tissue at super-low temperature IV. Effect of cooling and rewarming rates on survival. Plant Physiol.

[43] Strimbeck GR, Kjellsen TD, Schaberg PG, Murakami PF (2008). Dynamics of low-temperature acclimation in temperate and boreal conifer foliage in a mild winter climate. Tree Physiol.

[44] Strimbeck GR, Schaberg PG, Fossdal CG, Schröder WP, Kjellsen TD (2015). Extreme low temperature tolerance in woody plants. Front Plant Sci.

[45] Prásil I, Zámečník J (1990). Time course of electrolyte leakage from various samples killed by frost, liquid nitrogen or boiling. Biol Plant.

[46] van Gelderen DM, de Jong PC, Oterdoom HJ (1994). Maples of the world.

[47] Li J, Stukel M, Bussies P, Skinner K, Lemmon AR, Lemmon EM (2019). Maple phylogeny and biogeography inferred from phylogenomic data. J Syst Evol.

[48] Areces-Berazain F, Hinsinger DD, Strijk JS (2021). Genome-wide supermatrix analyses of maples (Acer, Sapindaceae) reveal recurring inter-continental migration, mass extinction, and rapid lineage divergence. Genomics.

[49] Grossman JJ. Evidence of constrained divergence and conservatism in climatic niches of the temperate maples (*Acer* L.). Forests 2021;12:535. 10.3390/f12050535

[50] Quamme HA (1986). Use of thermal analysis to measure freezing resistance of grape buds. Can J Plant Sci.

[51] Mills LJ, Ferguson JC, Keller M (2006). Cold-hardiness evaluation of grapevine buds and cane tissues. Am J Enol Vitic.

[52] Londo JP, Kovaleski AP (2017). Characterization of wild North American grapevine cold hardiness using differential thermal analysis. Am J Enol Vitic.

[53] Sakai A (1979). Freezing tolerance of evergreen and deciduous broad-leaved trees in Japan with reference to tree regions. Low Temp Sci Ser B, Biol Sci.

[54] R Core Team. R: A language and environment for statistical computing [Internet]. Vienna, Austria: R Foundation for Statistical Computing; 2020. https://www.r-project.org/.

[55] Ritz C, Baty F, Streibig JC, Gerhard D (2015). Dose-response analysis using R. PLoS ONE.

[56] de Mendiburu F. agricolae: Statistical procedures for agricultural research. 2020. https://cran.r-project.org/package=agricolae.

