# Supplementary material for: Standardization of electrolyte leakage data and a novel liquid nitrogen control improve measurements of cold hardiness in woody tissue
Source: Plant Methods. 2021 May 22;17:53. doi: 10.1186/s13007-021-00755-0 (PMC8140579; doi:10.1186/s13007-021-00755-0)
Supplement: Supplementary file 1 — Additional file 1: Figure S1. A) Native and naturalized distributions of the 12 maple study species, which are distributed across the North American (red/pink), European (green/yellow), and Asian (blue/purple) extent of the genus [49]. Star indicates the location of the Arnold Arboretum. B) Phylogenetic relatedness and ecological descriptions for the study species. Phylogeny and section designations adapted from [47,48]. [file 13007_2021_755_MOESM1_ESM.pdf]

A)

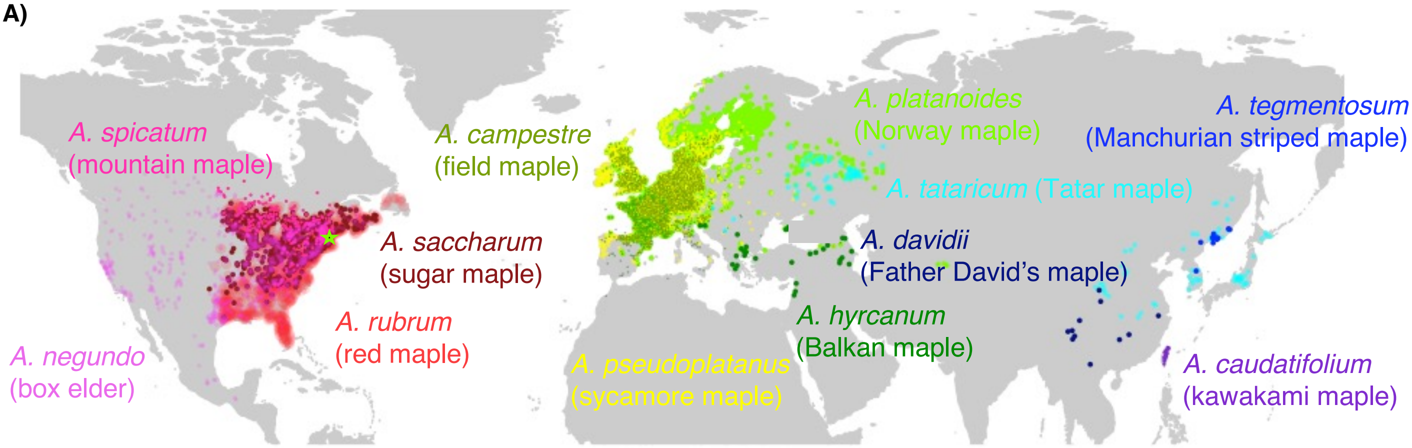

B)

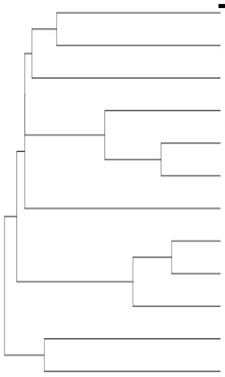

Species	Section	Region	Distribution	Habit
A. saccharum	Acer	Eastern N. America	Wide	Dominant
A. pseudoplatanus	Acer	Europe	Wide	Dominant
A. rubrum	Rubra	Eastern N. America	Wide	Dominant
A. platanoides	Platanoidea	Europe	Wide	Dominant
A. campestre	Platanoidea	Western Europe	Wide	Subdominant
A. hyrcanum	Acer	S. Europe/Asia Minor	Narrow	Shrub/Understory
A. tataricum	Ginnala	Pan-Asian	Wide	Ruderal
A. tegmentosum	Macrantha	East Asian	Narrow	Understory
A. caudatifolium	Macrantha	East Asian	Narrow	Understory
A. davidii	Macrantha	East Asian	Wide	Understory
A. spicatum	Spicata	Eastern N. America	Wide	Understory
A. negundo	Negundo	Pan-North American	Wide	Riparian

Additional file 1. A) Native and naturalized distributions of the 12 maple study species, which are distributed across the North American (red/pink), European (green/yellow), and Asian (blue/purple) extent of the genus [49]. Star indicates the location of the Arnold Arboretum. B) Phylogenetic relatedness and ecological descriptions for the study species. Phylogeny and section designations adapted from [47,48].
