# Supplementary material for: Standardization of electrolyte leakage data and a novel liquid nitrogen control improve measurements of cold hardiness in woody tissue
Source: Plant Methods. 2021 May 22;17:53. doi: 10.1186/s13007-021-00755-0 (PMC8140579; doi:10.1186/s13007-021-00755-0)
Supplement: Supplementary file 3 — Additional file 3: Figure S2. A) Validation of critical values from four approaches to modeling electrolyte leakage (as in Fig. 1) against visual estimates of freezing damage. Critical values reflect either 20%, 50%, or 80% electrolyte leakage (rows) or visual damage (columns). The rightmost column indicates lowest survival temperature (LST), the lowest temperature at which stems experienced < 50% damage. Pie wedge size indicates correlation. B) 50% electrolyte leakage values using the Limlogistic approach (orange box) best predicted visual damage in the 40-60% damage range. [file 13007_2021_755_MOESM3_ESM.pdf]

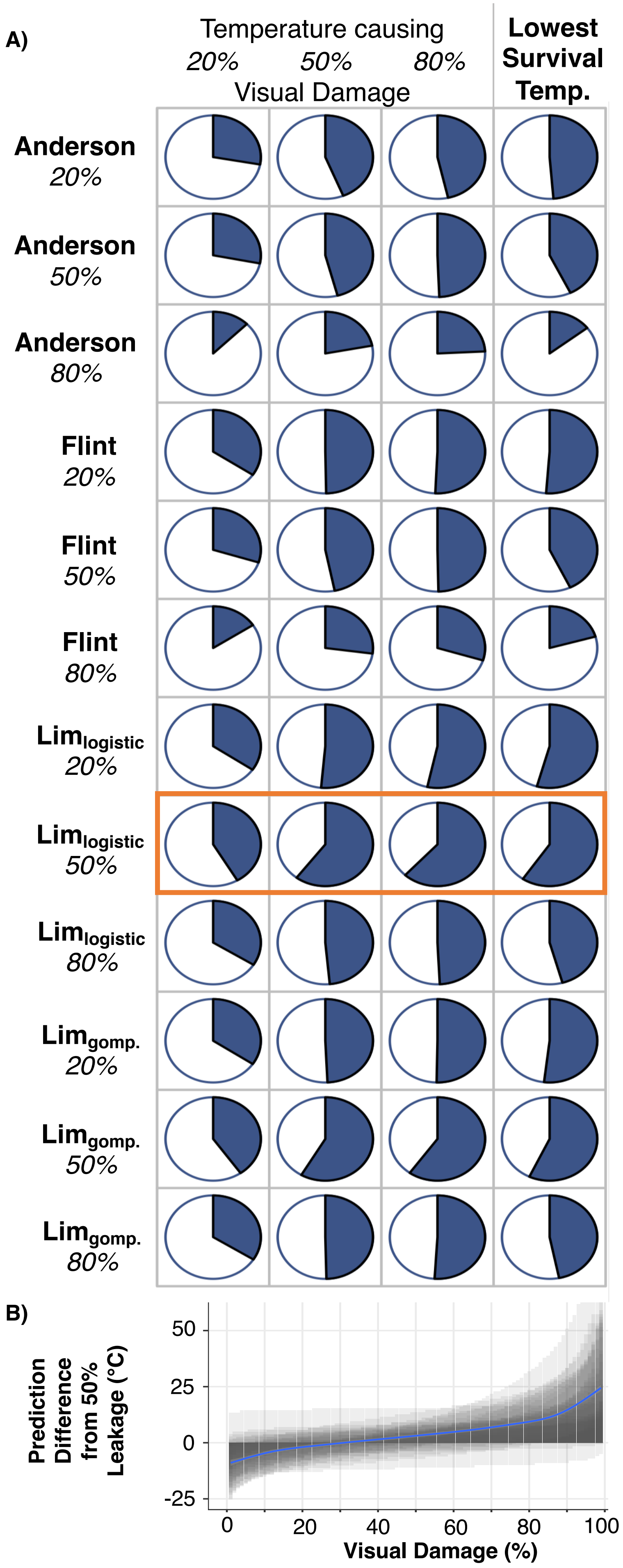

Additional file 3. A) Validation of critical values from four approaches to modeling electrolyte leakage (as in Fig. 1) against visual estimates of freezing damage. Critical values reflect either 20%, 50%, or 80% electrolyte leakage (rows) or visual damage (columns). The rightmost column indicates lowest survival temperature (LST; Sakai et al. 1986), the lowest temperature at which stems experienced < 50% damage. Pie wedge size indicate correlations. B) 50% electrolyte leakage values using the $Lim_{logistic}$ approach (orange box) best predicted visual damage in the 40-60% damage range.
