# Supplementary material for: Standardization of electrolyte leakage data and a novel liquid nitrogen control improve measurements of cold hardiness in woody tissue
Source: Plant Methods. 2021 May 22;17:53. doi: 10.1186/s13007-021-00755-0 (PMC8140579; doi:10.1186/s13007-021-00755-0)
Supplement: Supplementary file 4 — Additional file 4: Figure S3. Critical electrolyte leakage (estimated using the Limlogistic approach) best approximates 50% visual damage when leakage is between 50 and 80%. Bias, though, is lowest from 20 to 50% leakage. Color-coding indicates species (see bottom left panel). Error reflects variation among genotypes of a given species. [file 13007_2021_755_MOESM4_ESM.pdf]

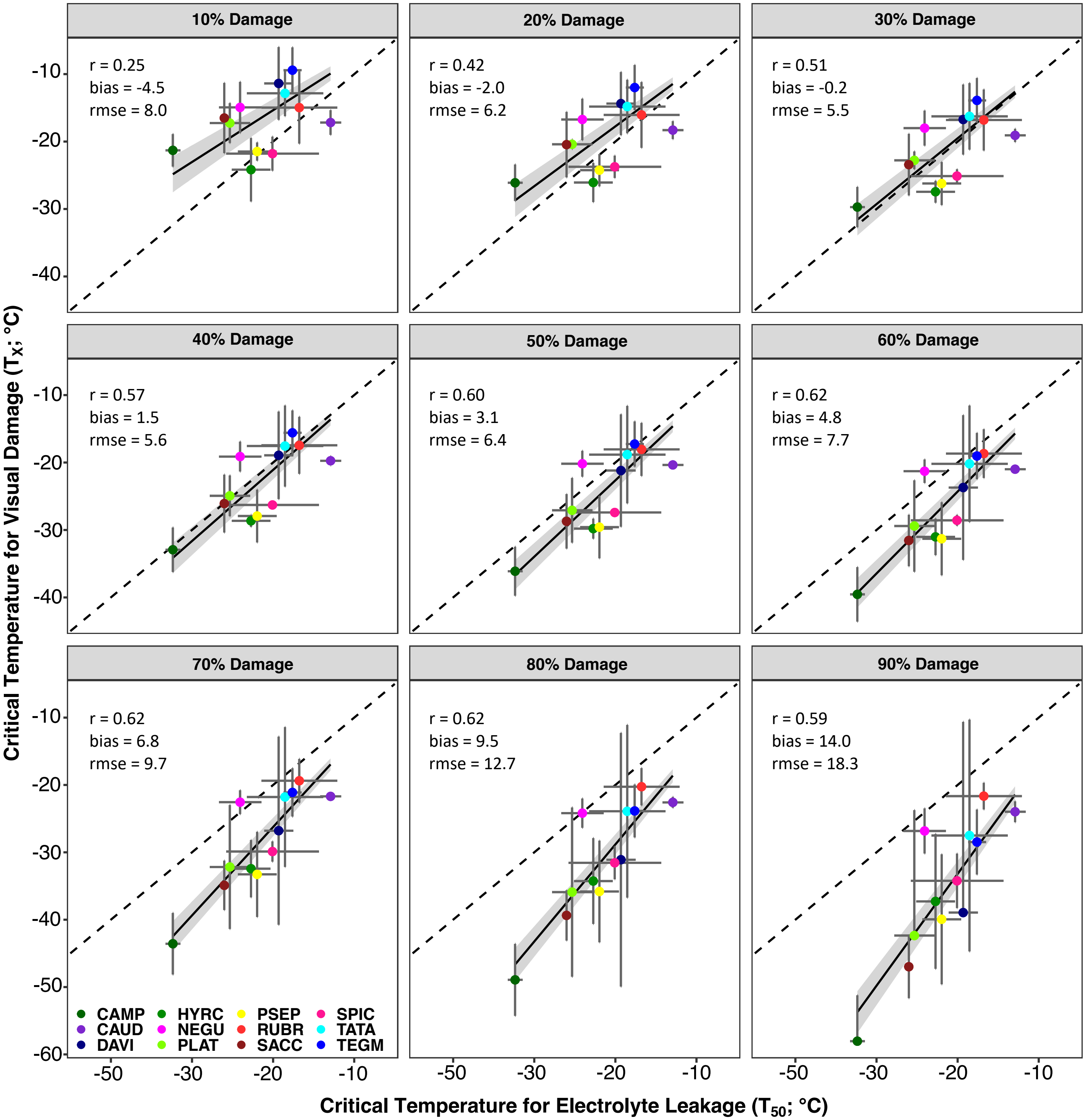

Additional file 4. Critical electrolyte leakage (estimated using the $Lim_{logistic}$ approach) best approximates 50% visual damage when leakage is between 50 and 80%. Bias, though, is lowest from 20 to 50% leakage. Color-coding indicates species (see bottom left panel). Error reflects variation among genotypes of a given species.
