# Supplementary material for: Standardization of electrolyte leakage data and a novel liquid nitrogen control improve measurements of cold hardiness in woody tissue
Source: Plant Methods. 2021 May 22;17:53. doi: 10.1186/s13007-021-00755-0 (PMC8140579; doi:10.1186/s13007-021-00755-0)
Supplement: Supplementary file 5 — Additional file 5: Figure S4. When a boiling standard is used, electrolyte leakage values derived using different curve-fitting procedures (e.g. Anderson vs. Limlogistic vs. Flint approaches) are not comparable above ~25% leakage (A vs. B). However, use of a liquid nitrogen standard makes outputs of these two routines more comparable (C vs. D). Grey bar indicates a range of values within 15% of the 1:1 line. [file 13007_2021_755_MOESM5_ESM.pdf]

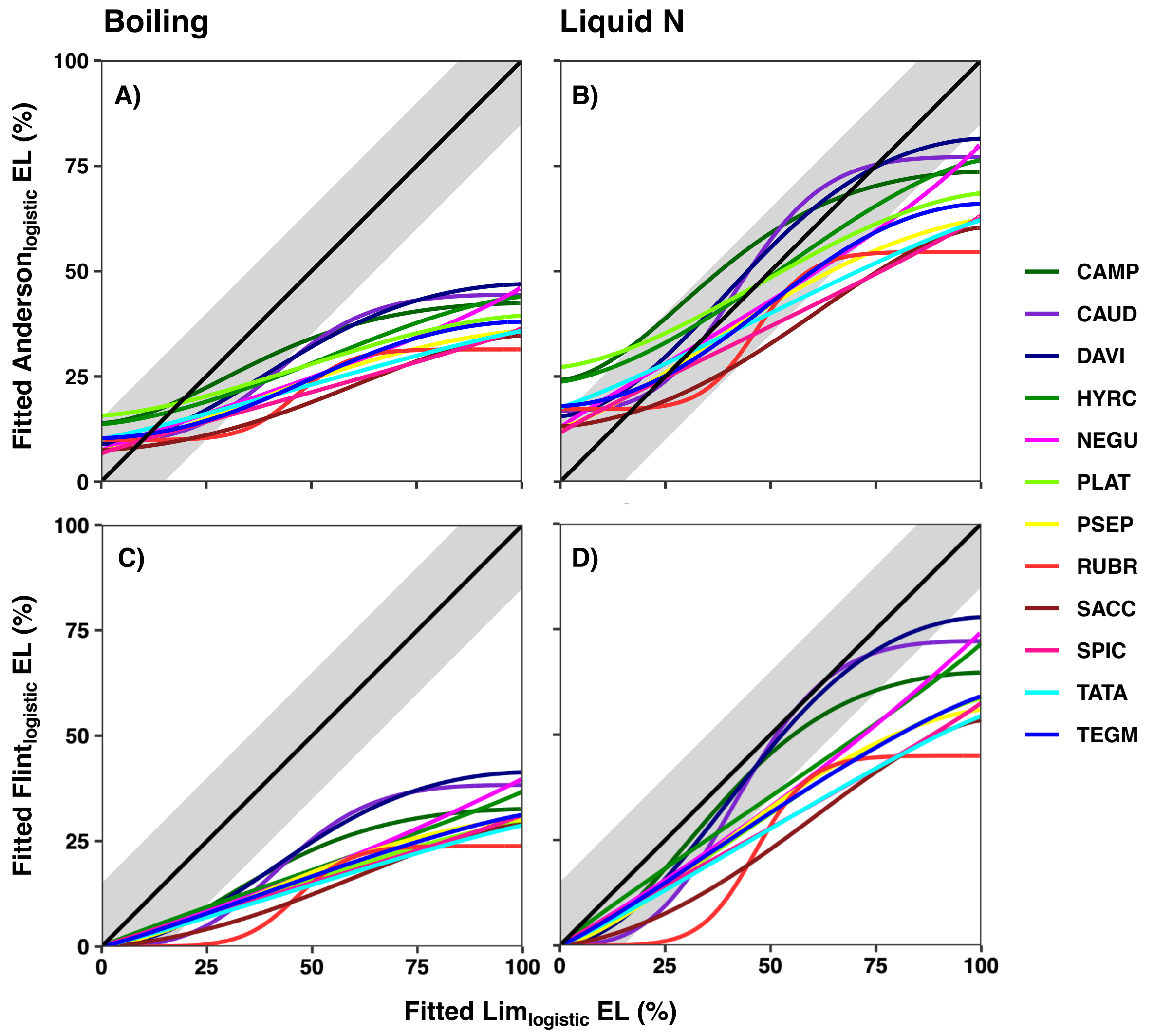

Additional file 5. When a boiling standard is used, electrolyte leakage values derived using different curve-fitting procedures (e.g. Anderson vs. Limlogistic vs. Flint approaches) are not comparable above ~25% leakage (A vs. C). However, use of a liquid nitrogen standard makes outputs of these two routines more comparable (B vs. D). Grey bar indicates a range of values within 15% of the 1:1 line.
