# Supplementary material for: Standardization of electrolyte leakage data and a novel liquid nitrogen control improve measurements of cold hardiness in woody tissue
Source: Plant Methods. 2021 May 22;17:53. doi: 10.1186/s13007-021-00755-0 (PMC8140579; doi:10.1186/s13007-021-00755-0)
Supplement: Supplementary file 6 — Additional file 6: Figure S5. A) Electrolyte leakage increased gradually over seven days following experimental freezing (light blue), but not exposure to a boiling or liquid nitrogen control (turquoise), with no evidence of a significant difference when conductivity was measured over the first 48 hours after freezing. Lowercase letters indicate significant differences in conductivity measured at different time points at the 0.05 level based on models reported in Additional file 2C panels A (light blue, a-c) and B (turquoise, d-e). B) As a result of this pattern, estimates of critical values for cold hardiness (T50) are consistent and reflect species differences when samples were incubated for one or two days, but not when they were incubated for 5 or 7 days (Additional file 2D). [file 13007_2021_755_MOESM6_ESM.pdf]

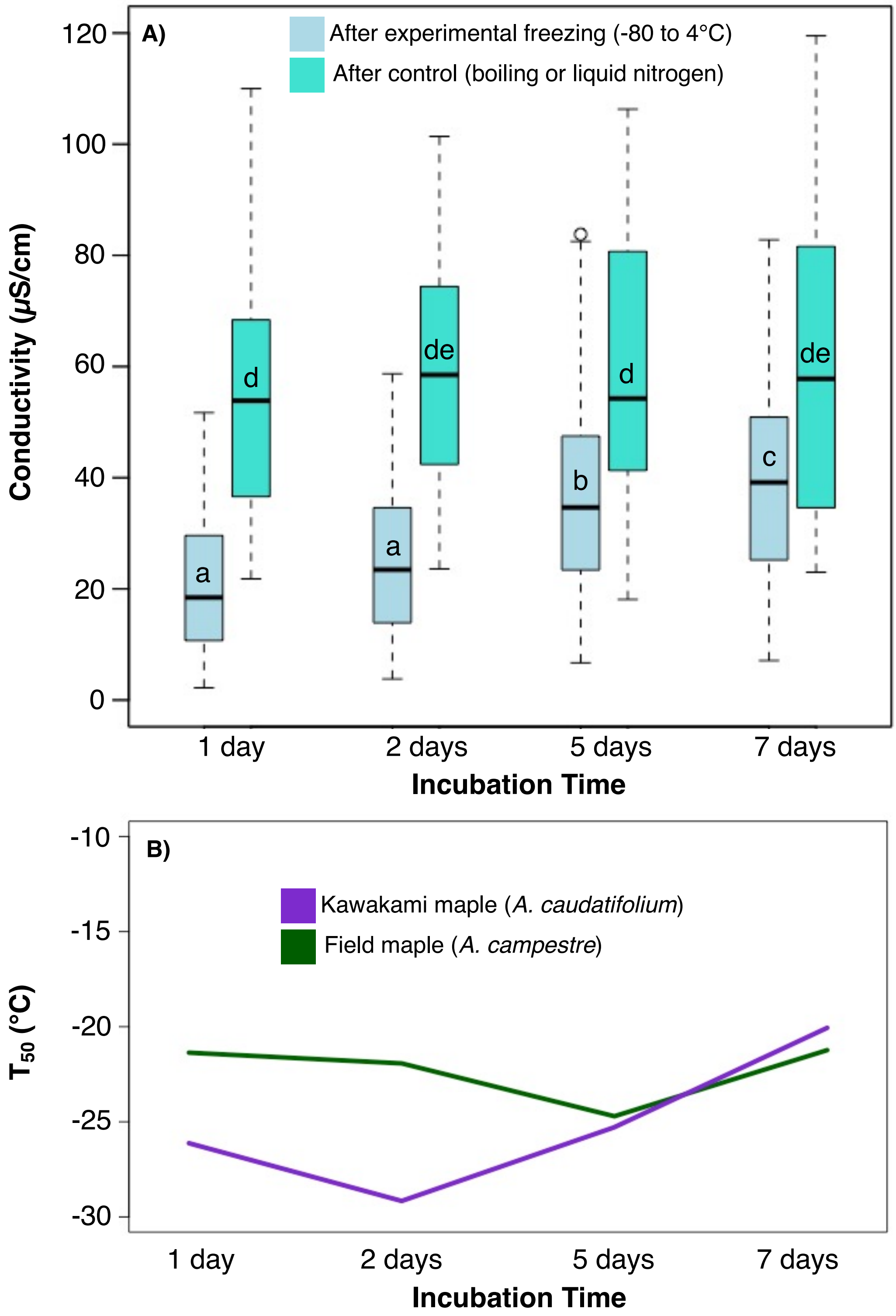

Additional file 6. A) Electrolyte leakage increased gradually over seven days following experimental freezing (light blue), but not exposure to a boiling or liquid nitrogen control (turquoise), with no evidence of a significant difference when conductivity was measured over the first 48 hours after freezing. Lowercase letters indicate significant differences in conductivity measured at different time points at the 0.05 level based on models reported in Additional file 2C panels A (light blue, a-c) and B (turquoise, d-e). B) As a result of this pattern, estimates of critical values for cold hardiness (T_{50}) are consistent and reflect species differences when samples were incubated for one or two days, but not when they were incubated for 5 or 7 days (Additional file 2D).
