# Supplementary material for: Standardization of electrolyte leakage data and a novel liquid nitrogen control improve measurements of cold hardiness in woody tissue
Source: Plant Methods. 2021 May 22;17:53. doi: 10.1186/s13007-021-00755-0 (PMC8140579; doi:10.1186/s13007-021-00755-0)

Additional file 7. Stem segments incubated for longer than five days following control treatment (boiling or liquid nitrogen immersion) tended to deteriorate, showing evidence of microbial growth.

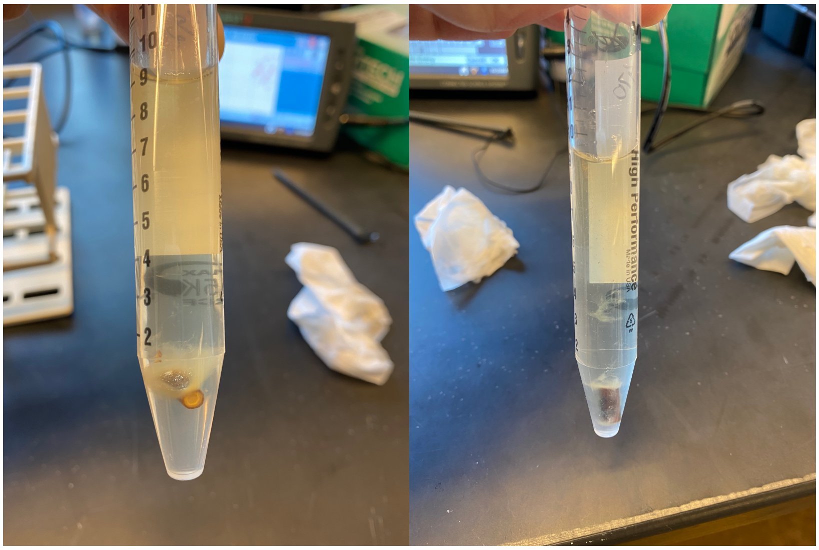

Supplement: Supplementary file 7 — Additional file 7: Figure S6. Stem segments incubated for longer than five days following control treatment (boiling or liquid nitrogen immersion) tended to deteriorate, showing evidence of microbial growth. [file 13007_2021_755_MOESM7_ESM.pdf]
